# Predictive value of DNA methylation patterns in AML patients treated with an azacytidine containing induction regimen

**DOI:** 10.1186/s13148-023-01580-z

**Published:** 2023-10-26

**Authors:** Maximilian Schmutz, Manuela Zucknick, Richard F. Schlenk, Daniel Mertens, Axel Benner, Dieter Weichenhan, Oliver Mücke, Konstanze Döhner, Christoph Plass, Lars Bullinger, Rainer Claus

**Affiliations:** 1 Hematology and Oncology, Medical Faculty, University of Augsburg, Stenglinstr. 2, 86156 Augsburg, Germany; 2 Division of Cancer Epigenomics, German Cancer Research Center (DKFZ), Heidelberg, Germany; 3 Oslo Centre for Biostatistics and Epidemiology, University of Oslo, Oslo, Norway; 4 NCT-Trial Center, National Center of Tumor Diseases, German Cancer Research Center, Heidelberg University Hospital, Heidelberg, Germany; 5 Department of Internal Medicine V, Heidelberg University Hospital, Heidelberg, Germany; 6 Cooperation Unit “Mechanisms of Leukemogenesis”, German Cancer Research Center, Heidelberg, Germany; 7 Division of Chronic Lymphocytic Leukemia, Department of Internal Medicine III, Ulm University Medical Center, Ulm, Germany; 8 Division of Biostatistics, German Cancer Research Center (DKFZ), Heidelberg, Germany; 9 Department of Internal Medicine III, University Hospital of Ulm, Ulm, Germany; 10 German Cancer Consortium (DKTK), Partner Site Berlin, Berlin, Germany; 11 Department of Hematology, Oncology, and Cancer Immunology, Campus Virchow Klinikum, Berlin, Charité-Universitätsmedizin Berlin, Corporate Member of Freie Universität Berlin, Humboldt-Universität zu Berlin, Berlin, Germany; 12 Pathology, Medical Faculty, University of Augsburg, Augsburg, Germany

**Keywords:** DNA-methylation, Epigenetics, HMA-treatment, Predictive biomarker, Predictive signature, DNA methylation patterns, AML, Azacytidine

## Abstract

**Background:**

Acute myeloid leukemia (AML) is a heterogeneous disease with a poor prognosis. Dysregulation of the epigenetic machinery is a significant contributor to disease development. Some AML patients benefit from treatment with hypomethylating agents (HMAs), but no predictive biomarkers for therapy response exist. Here, we investigated whether unbiased genome-wide assessment of pre-treatment DNA-methylation profiles in AML bone marrow blasts can help to identify patients who will achieve a remission after an azacytidine-containing induction regimen.

**Results:**

A total of *n* = 155 patients with newly diagnosed AML treated in the AMLSG 12-09 trial were randomly assigned to a screening and a refinement and validation cohort. The cohorts were divided according to azacytidine-containing induction regimens and response status. Methylation status was assessed for 664,227 500-bp-regions using methyl-CpG immunoprecipitation-seq, resulting in 1755 differentially methylated regions (DMRs). Top regions were distilled and included genes such as *WNT10A* and *GATA3*. 80% of regions identified as a hit were represented on HumanMethlyation 450k Bead Chips. Quantitative methylation analysis confirmed 90% of these regions (36 of 40 DMRs). A classifier was trained using penalized logistic regression and fivefold cross validation containing 17 CpGs. Validation based on mass spectra generated by MALDI-TOF failed (AUC 0.59). However, discriminative ability was maintained by adding neighboring CpGs. A recomposed classifier with 12 CpGs resulted in an AUC of 0.77. When evaluated in the non-azacytidine containing group, the AUC was 0.76.

**Conclusions:**

Our analysis evaluated the value of a whole genome methyl-CpG screening assay for the identification of informative methylation changes. We also compared the informative content and discriminatory power of regions and single CpGs for predicting response to therapy. The relevance of the identified DMRs is supported by their association with key regulatory processes of oncogenic transformation and support the idea of relevant DMRs being enriched at distinct loci rather than evenly distribution across the genome.

Predictive response to therapy could be established but lacked specificity for treatment with azacytidine. Our results suggest that a predictive epigenotype carries its methylation information at a complex, genome-wide level, that is confined to regions, rather than to single CpGs. With increasing application of combinatorial regimens, response prediction may become even more complicated.

**Supplementary Information:**

The online version contains supplementary material available at 10.1186/s13148-023-01580-z.

## Introduction

Acute myeloid leukemia (AML) is a biologically and clinically heterogeneous disease characterized by clonal expansion of undifferentiated myeloid precursors and consequently by impaired hematopoiesis. Despite recent advances in therapeutic interventions and supportive care, the prognosis remains poor, especially for elderly patients [1, 2].

Distinct recurrent cytogenetic and molecular genetic aberrations have been shown to define AML pathophysiology and to harbor considerable prognostic relevance [3–5]. Disturbances of epigenetic mechanisms including alterations of DNA methylation patterns significantly contribute to AML development and are tightly associated with patterns of genetic aberrations such as mutations of epigenetic modifier genes (e. g. *IDH1*, *IDH2, DNMT3A, TET2* etc.) and others such as *CEBPA*, *NPM1*, and *FLT3* [6–9]. Genomewide epigenetic profiling has revealed DNA methylation driven AML subclassifications, some of which correlate with known genetic aberrations but also include novel subgroups [7, 8]. Recently, a comprehensive analysis has shown that AML can be subdivided into different epitypes based on DNA methylation, which can be associated with genetic aberrations and attributed to blockage of differentiation at specific stages of myeloid differentiation. [10]. Numerous aberrantly hyper- or hypomethylated genomic regions possess significant prognostic relevance and have been proposed as biomarkers [7, 11, 12].

DNA-hypomethylating agents (HMAs), e.g., azacytidine (AZA) and its deoxy derivative 5-aza-2’-deoxycytidine (DAC), which exert hypomethylating effects by passive incorporation into DNA during S phase and by covalently binding the maintenance methyltransferase DNMT1 [13], have been tested for in vivo demethylation and have become an accepted standard treatment regimens for MDS and AML in elderly patients or in patients considered unsuitable for intensive chemotherapy [2, 14, 15]. Azacytidine, both alone and in combination, e. g. with the BCL2 inhibitor Venetoclax, has been shown to be highly active in newly diagnosed AML and molecularly defined subsets of relapsed or refractory AML [16–18]. However, it remains unclear which patients will ultimately respond to HMAs [19–21]. Even for responders, development of resistance within a year is not an uncommon event, irrespective of superior overall survival and high rates of remission introduced by the novel combination of HMAs with BCL2-inhibition [17, 22–25]. This development underscores the urgent need to identify reliable predictors of outcomes and particularly to identify predictive biomarkers for drugs targeting the epigenome [26].

Biomarkers for response prediction to demethylating agents in MDS and AML are the subject of ongoing research efforts [27]. However, studies on epigenetic changes in AML have not yet established a strong correlation between response to HMA and baseline DNA methylation profiles, let alone developed a predictive toolkit that can be translated and used in routine clinical practice [28–32].

Several molecular markers with potential response prediction have been identified, including pharmacologic factors, clinical or cytogenetic parameters, DNA methylation—and its dynamics upon HMA treatment—as well as molecular alterations and changes in gene expression [33–36]. Additional file 3: Table 1 provides an overview of research work intended at identifying predictive molecular markers for treatment with DNA-methyltransferase-inhibitors (DNMTi) in AML, MDS and selected hematologic malignancies: To date, predictive methylation-specific biomarkers associated with AML have neither been successfully established nor introduced into clinical practice. Signatures of prognostic value have been shown to harbor predictive information, either in AML, MDS or MDS/MPN overlap but were either not validated in an independent cohort or derived from a very small sample set and could not be reproduced so far [37, 38]. Among other factors, the limited selection of genomic regions, e.g., strict focus on promoter methylation, has been consistently cited as a reason for failure in developing a robust predictive classifier.

**Table 1:** Baseline patient and disease characteristics

	Total (*n* = 155)	Screening cohort (*n* = 58)	Validation cohort (*n* = 97)	*p*
Age in years median (IQR)	60.50 (50.26, 68.49)	63.46 (50.32, 72.01)	59.69 (50.24, 67.84)	0.125
Sex, *n* (%)				0.32
female	69 (45)	29 (51)	40 (41)	
male	85 (55)	28 (49)	57 (59)	
WBC 10^9^/L (*n* = 152) median (IQR)	5.30 (2.05, 25.80)	4.70 (1.70, 19.80)	5.85 (2.50, 33.02)	0.325
PB blast % (*n* = 141) median (IQR)	21.50 (4.00, 53.50)	16.00 (4.00, 53.00)	23.00 (4.50, 54.00)	0.535
BM blast % (*n* = 146) median (IQR)	63.00 (40.00, 80.00)	60.00 (41.50, 80.50)	70.00 (40.25, 80.00)	0.507
Cytogenetics, (*n* = 135)				
CN-AML, *n* (%)	56 (42)	21 (42)	35 (42)	1
complex.karyotype, *n* (%)	26 (19)	9 (18)	17 (20)	0.927
t(11q23), *n* (%)	8 (6)	3 (6)	5 (6)	1
del(5q)/-5, *n* (%)	5 (4)	1 (2)	4 (5)	0.65
inv(3)/t(3;3), *n* (%)	2 (1)	1 (2)	1 (1)	1
other, *n* (%)	37 (28)	15 (30)	22 (26)	0.782
Mutated *RUNX1*, *n* (%)	32 (21)	15 (27)	17 (18)	0.239
Mutated *IDH1*, *n* (%)	12 (8)	5 (9)	7 (7)	0.758
Mutated *IDH2*, *n* (%)	22 (15)	10 (18)	12 (13)	0.492
Mutated *DNMT3A*, *n* (%)	26 (17)	16 (29)	10 (10)	0.007
Mutated *ASXL1*, *n* (%)	23 (17)	8 (16)	15 (18)	0.969
Mutated *TP53*, *n* (%)	14 (10)	6 (12)	8 (9)	0.854

WBC white blood cell count, PB blast peripheral blood blast count, BM blast, bone marrow blast count, *CEBPA*, CCAAT/ enhancer-binding protein alpha, *DNMT3A* DNA methyltransferase 3A, *ASXL1* additional sex combs-like 1, *RUNX1* runt-related transcription factor 1, *IDH* Isocitrate dehydrogenase, *TP53* tumor protein P53

Here we asked if unbiased genome-wide assessment of pre-treatment DNA-methylation profiles in AML bone marrow blasts could aid in identifying patients who will achieve a remission upon azacytidine-containing therapy or who will fail induction therapy. Bone marrow samples were obtained from the AMLSG 12-09 trial. This randomized, controlled, prospective, multi-institutional and controlled phase-II trial evaluated the incorporation of the hypomethylating agent azacytidine into intensive induction therapy as a substitute for cytarabine. The patient population was lacking molecularly defined subtypes which would allow for genotype specific therapy approaches—mainly as participants in competing trials—such as patients with mutated *NPM1*, AML with *FLT3*-ITD, *PML-RARA* fusion, and CBF-AML. This resulted in a selection of patients with more high-risk disease features [39]. The results of this trial did not generally support the substitution of cytarabine by azacytidine in intensive induction therapy. Moreover, a predictive biomarker to identify patients who may benefit from the additional administration of an HMA has yet to be developed.

## Results

### Baseline characteristics of patients

A total of *n* = 155 patients with newly diagnosed AML treated within the AMLSG 12-09 trial and with available pre-treatment bone marrow samples were randomly assigned to a screening (*n* = 58) and a refinement and validation cohort (*n* = 97). The median age in the screening cohort was 63 years (range 20 to 78) and 60 years in the refinement and validation cohort (range 19 to 82). 95% and 94% of patients were younger than 75 years in the two cohorts, respectively. Sex distribution was imbalanced between both cohorts with 52% female patients in the screening cohort and 41% in the refinement and validation cohort.

**Fig. 1:**
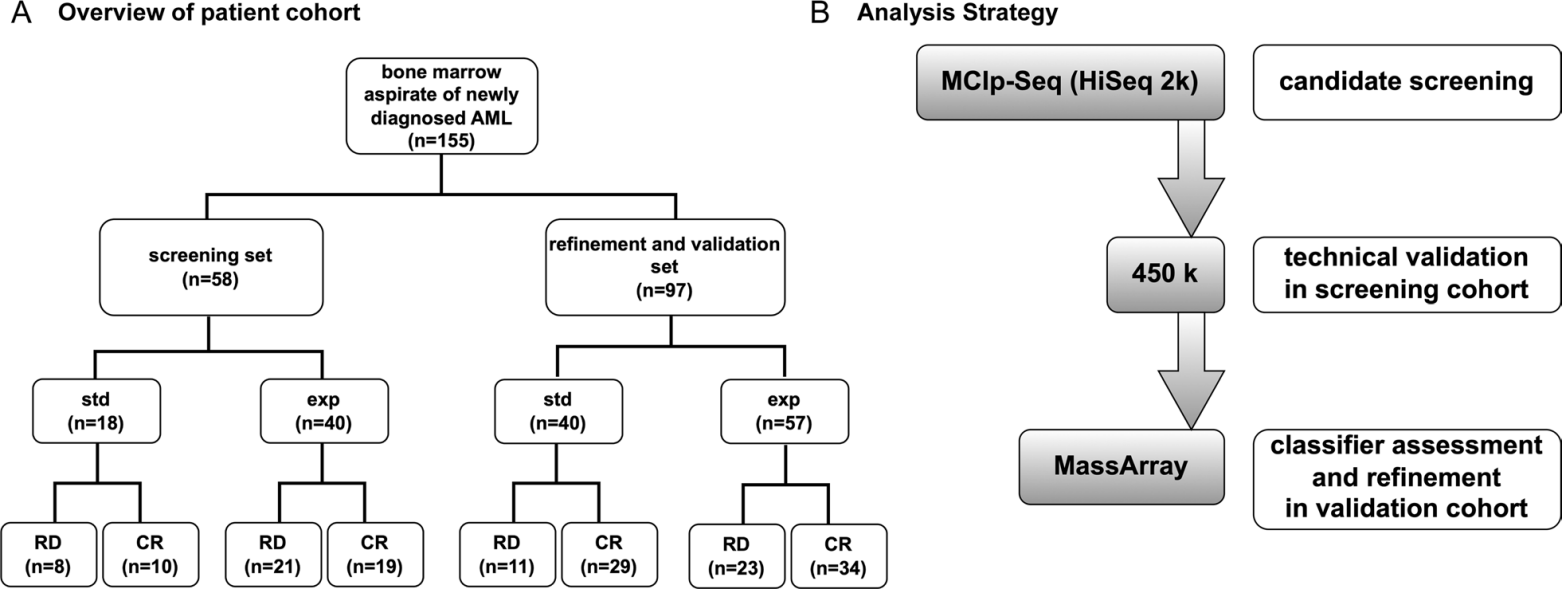
Analysis overview. **A** Overview of analysis steps based on DNA isolated from mononuclear cells from each pretreatment bone marrow aspirate from a subset of 155 AML samples derived from the AMLSG 12-09 trial. Global, genome-wide methylation status of a training set was analysed via MCIp followed by NGS-analysis on the HiSeq 2k platform. Differentially methylated regions were derived and ranked according to *p*-values and effect size. Methylation levels within a set of top regions were validated via 450k analysis at single CpG resolution and used to generate a classifier. **B** The validation cohort consisted of an independent subset of patients derived from the AMLSG 12-09 collective. Methylation status of the classifier contained CpGs was analysed via MassARRAY assay and used for validation. CR was defined as non-detectability of evidence for disease both cytomorphologically and via immunophenotyping in peripheral blood smear and bone marrow aspirate as well as via molecular genetics. *AML* acute myeloid leukemia; *DMR* differentially methylated regions; *MCIp* methyl-CpG immunoprecipitation; *HiSeq 2k* the HiSeq next-generation sequencing platform; *NGS* next generation sequencing; *450k* Infinium® HumanMethylation450 Bead Chip; *MassARRAY* a benchtop multiplex genetic analyzer utilizing Matrix assisted laser desorption/ionization; time-of-flight mass spectrometry; *std* standard therapy arm; *exp* experimental therapy arm; *CR* complete response; *RD* refractory disease

Out of 58 patients of the screening cohort, 18 received standard of care treatment (STD) and 40 patients received experimental treatment (EXP) comprising AZA as substitute for cytarabine (araC) (Fig. 1A).

Within the STD arm, 10 patients achieved complete remission (CR) and 8 patients had incomplete remission/induction failure (referred to as refractory disease, RD). For the EXP arm, CR was achieved in 19 patients and RD in 21 patients, respectively.

Within the refinement and validation cohort, out of 97 patients, 40 were treated in the STD arm and 57 in the EXP arm. CR within STD treatment was achieved in 29 patients, while 11 patients had RD. For 34 patients with EXP therapy, CR was observed, while 23 patients had RD.

In the screening cohort (refinement and validation cohort, correspondingly in brackets), median white blood cell count was 6 G/l with a range of 0.6–155 G/l (6 G/l; range 1–214 G/l), median peripheral blood blast count was 17.5% with a range of 0–97% (23%; range 0–97% and median bone marrow blasts were 60.5% with a range of 15–100 (70%; range 10–100%) (Table 1).

Cytogenetic analysis revealed 21 (35) patients with a normal karyotype (CN), 9 (17) patients with a complex karyotype (CK), 1 (4) patient with a 5q-minus-syndrome or loss of chromosome 5 (del(5q)/-5), 2 (1) with a MECOM rearrangement (inv(3)/t(3;3)), 3 (5) patients with a translocation 11q23 and 15 (22) patients with a karyotype not otherwise specified. In total, cytogenetic information was missing in 7 (13) cases (Table 1). Recurrent aberrations leading to genotype-specific therapeutic approaches (e. g. *FLT3*-ITD) at the time of inclusion in the study were excluded according to the protocol. Mutational status in a panel of seven genes recurrently mutated in myeloid neoplasia (*TP53, ASXL1, DNMT3A, RUNX1, IDH1, IDH2, TET*2) including regulators of the epigenotype did not correlate with AZA response. However, a significant difference in the number of DNMT3A mutations was observed between the screening and validation cohort. Moreover, there was no association of cytogenetic subgrouping or mutations in epigenetic modifier genes with therapy response (Additional file 2: Fig. 1). To assess the impact of the mutations on the overall methylation landscape in the screening cohort, we performed unsupervised clustering of the 1.000 and 10.000 most variable 500 bp bins of the MCIp analysis (Additional file 2: Fig. 2). Moderate clustering with discrete methylation patterns of *DNMT3A* and *IDH2* was evident, whereas *IDH1* and *ASXL1* did not appear to have a significant impact. The major clusters of this unsupervised hierarchical clustering were not primarily driven by the mutations in the epigenetic modifier genes. In addition, we reviewed the distribution of mutations in the epigenetic modifier genes as well as the distribution of cytogenetic aberrations in our potential top DMRs between responding and refractory patients after evaluation for differential methylation (Additional file 2: Fig. 3). There was no segregation with response. Standard and experimental treatment arms within the screening cohort did not differ significantly regarding clinical characteristics except for bone marrow blast counts which were significantly higher in the exp-arm (66.5% versus 50.0%) than in the std-arm (*p* = 0.02) (Additional file 4: Table 2).

**Fig. 2:**
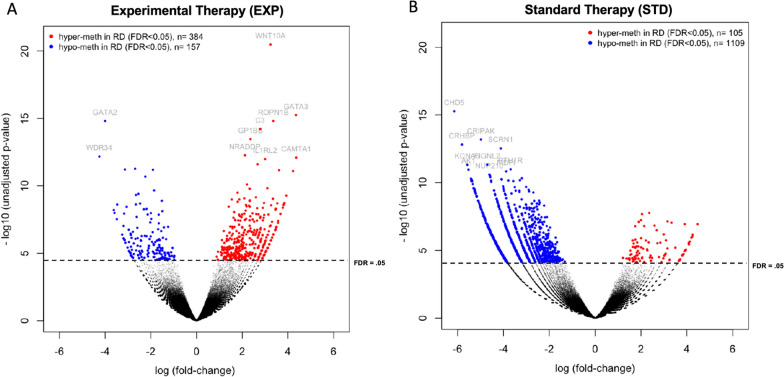
Significant Baseline DNA Methylation Differences reveal less methylation in refractory disease of AZA containing treatment regimens. **A** Volcano Plot illustrating methylation differences between AZA-sensitive and AZA-resistant (Experimental Therapy) as well as **B** induction sensitive and induction resistant patients (Standard Therapy). Mean methylation difference between the 2 groups is represented on the x axis and statistical significance (-log10 unadjusted *p*-value) on the y axis. Negative binomial distribution-based testing with edgeR identified 1755 DMRs, indicated by red and blue dots (FDR < 5% with adjustment for multiple testing)

**Fig. 3:**
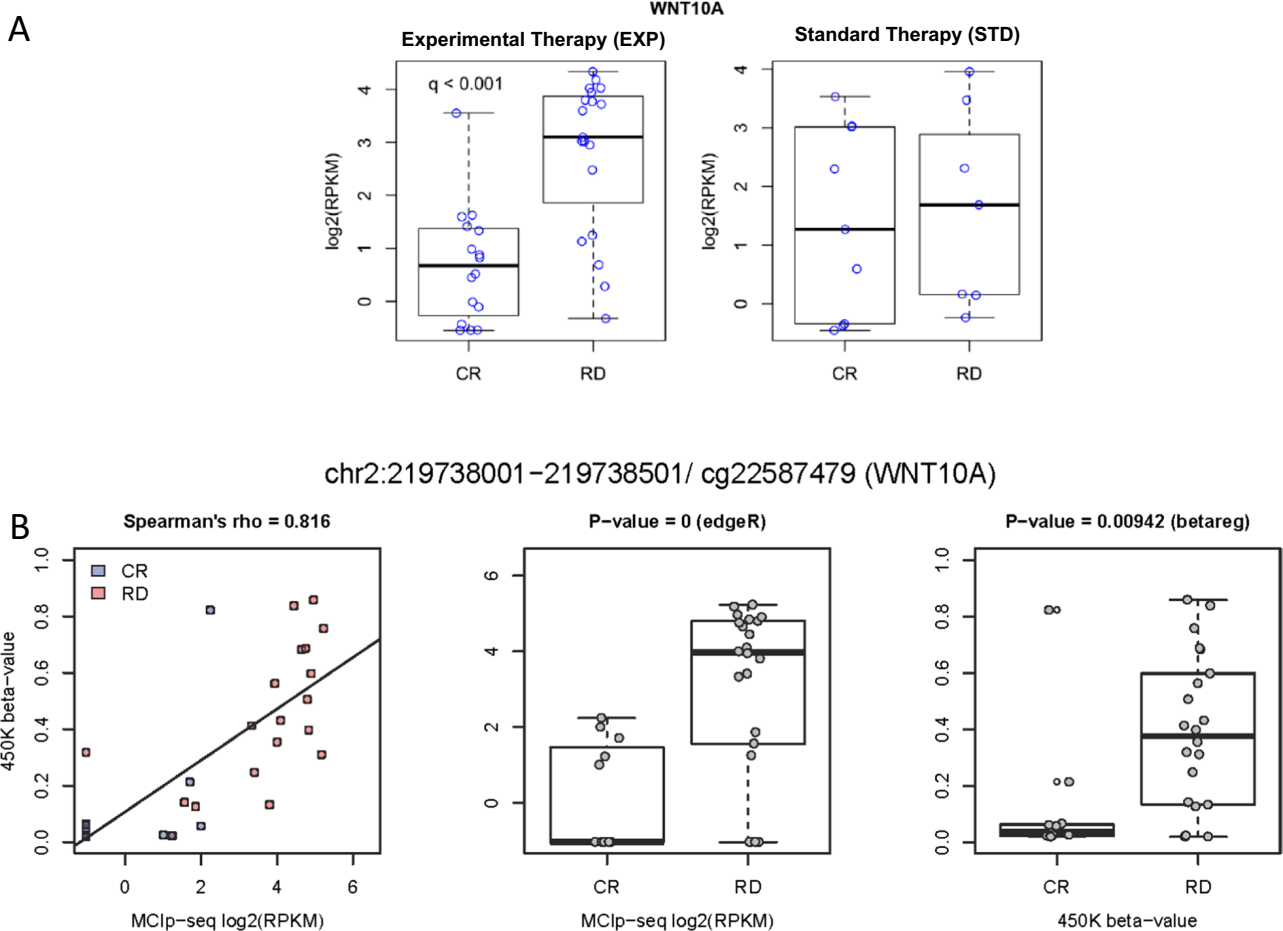
Technical Validation of Differentially Methylated Regions. **A** Selection of EdgeR-based testing results for differential methylation between responders and non-responders both in EXT and STD arm, prior to validation. **B** Validation criteria are exemplarily illustrated for the 500 bp region assigned to *WNT10A* and its corresponding probe cg22587479. For this probe, a strong and distinct correlation between beta values and RPKM exists (Spearman’s rank correlation coefficient > 0.8). Differences in beta regression levels between resp. and non-resp. patients showed statistical significance and overall methylation differences showed congruency in the change between modalities, i.e. hypermethylation in patients with refractory disease both in the MCIp-seq and 450k assay. *CR* Complete Response; *RD* Refractory Disease; *RPKM* Reads per kilobase per million mapped reads

### Genomewide DNA methylation screening within the screening cohort

For the development of a predictive classifier based on genome wide differential DNA methylation patterns, methyl-CpG immunoprecipitation-seq (MCIp-seq) of BM PBMC from AML patients in the screening cohort (*n* = 58) either treated within the STD or EXP arm was performed (Fig. 1B). Seven samples from the STD and EXP arm with very low read counts (mean read count < 1.0 × 10^6 reads) were flagged as outliers and removed from further analyses (Additional file 2: Fig. 4). A total of 51 samples remained in the screening cohort.

**Fig. 4:**
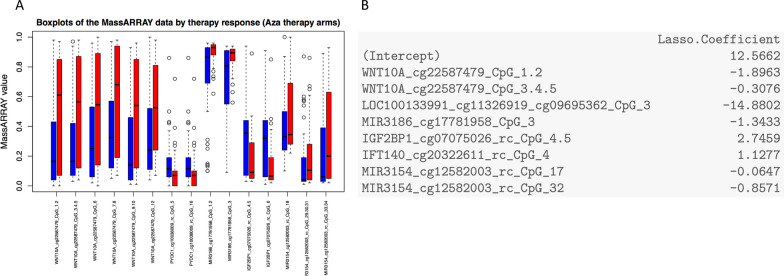
Significantly differentially methylated CpGs in close proximity to CpGs from original classifier define a recomposed classifier within an independent validation cohort. **A** Box plots for significant differences in methylation levels between responders (blue color) and non-responders (red color) as assessed by non-parametric wilcoxon rank sum tests in the EXP arm. Methylation levels were determined by MassARRAY assay. **B** Elements of a recomposed classifier based on a penalized likelihood regression model

Informative differential CpG methylation was retrieved for 664,227 (14%) out of more than 6 × 10^6 genomic bins enriched for high methylation by removing all bins with no reads across all or all but one sample.

Principal component analysis did not show formation of sample clusters (Additional file 2: Fig. 4) and components of variance did not display major effects in DNA methylation variance that allowed to reliably separate between treatment groups or response status. Overall, differences in variance distribution across principal components were subtle (data not shown).

Differential methylation analysis between responding and non-responding patients revealed twice as many regions with a significantly differing positive log-fold change (*n* = 384 vs. *n* = 157) in patients within the experimental treatment arm (Fig. 2A) indicating a higher fraction of hypermethylated regions in patients with refractory disease [40]. In the standard treatment arm, predominantly negative log-fold changes were observed within the group of responders (factor of 9.5 with *n* = 1109 vs *n* = 105) (Fig. 2B).

Overall distribution of differentially methylated regions (DMR) (*n* = 5.7 × 10^6, comprising both the EXP- and STD-set after filtering for positive read counts across all samples) within the filtered set of genomic bins shows higher read counts in exons while the set of top DMRs shows higher proportions of read counts with an intergenic and intronic location (Additional file 2: Fig. 5).

**Fig. 5:**
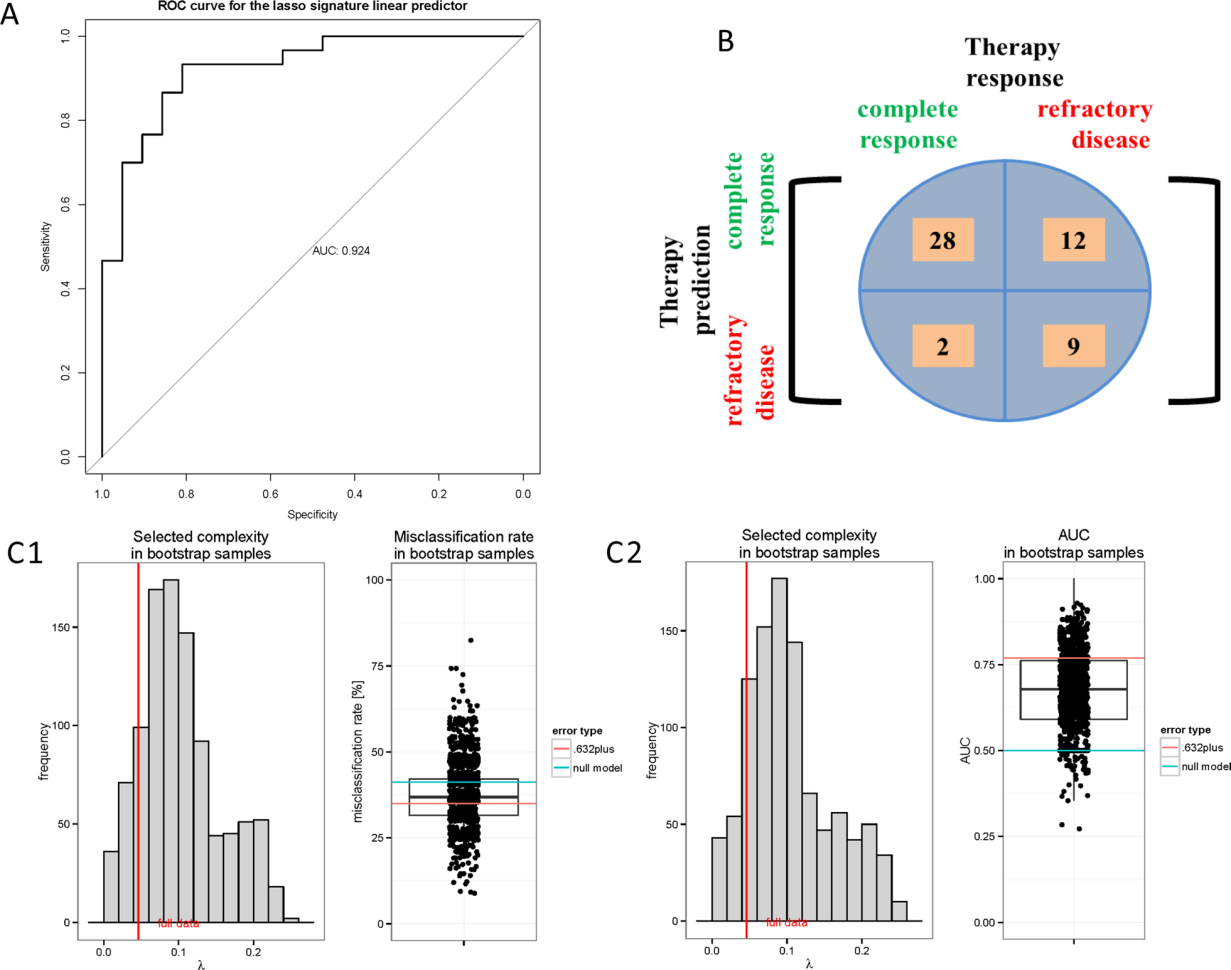
Quality assessment of the predictive model. **A** The AUC for the recomposed classifier is 0.924 and significantly improved over the previous version (AUC 0.59) resulting in a sensitivity of 93.3% and a specificity of 42.85% with a corresponding positive predictive (70%) and negative predictive value of 81.8% for the given results (**B**). **C** Final assessment via correction for multiple testing with .0632 + bootstrap resampling estimates reveal a misclassification error of 35% (**C1**) and a bootstrap estimation for the AUC of 0.77 (**C2**). *ROC* Receiver Operating Characteristic Curve. *AUC* Area under the curve. *λ* lambda, lasso penalty value

### Identification of specific response prediction signature for the 5-azacytidine containing treatment arm (EXP)

In total, considering both positive and negative log-fold changes, 1755 DMRs were identified at a false discovery rate (FDR) of 5% (541 in EXP and 1214 in STD arm) with adjustment for multiple testing. Identified DMRs were ranked according to q-values, i.e. adjusted *p*-values after multiple testing, and grouped into a top list. 50 candidates were chosen for validation based on the following criteria: q-value ranking, effect size and consistency of differential methylation in either treatment group. Effect size was set to include a read count difference of at least 2.5-fold in a consistent fraction of at least 50% of samples in either treatment group. Regions found on chromosomes 3 and 11 were excluded from analysis, as patients with inv(3)/t(3;3) and a translocation 11q23 could artificially introduce differential methylation on screening via MCIp-seq. This restriction affected less than 5% of choices for the top list. Because of the slightly uneven gender distribution between screening and validation cohort, sex chromosomes were also excluded from the analysis.

To extract an AZA specific response signature, DMR sets identified both in the EXP and STD arm were checked for overlaps as these were considered to potentially indicate unspecific global or chemotherapy associated effects on differential methylation, rather than AZA specific effects. Within the chosen top list, there were no overlaps between both DMR sets. Additional file 5: Table 3 contains a list of all filtered and significant DMRs, identified within the EXP arm. *WNT10A* shows an exemplary top hit (Fig. 3A).

Furthermore, enrichment in the vicinity of transcriptional start sites (TSS) of identified top DMRs as compared to the overall, filtered bin set could be observed. Moreover, GC content distribution in the set of top DMRs showed distinct skewing at a GC content level between 60 and 70% but was otherwise comparable to the entire genome, therefore indicating overrepresentation of higher GC content in the set of top hits (Additional file 2: Fig. 6). The first top DMRs included the genes *WNT10A*, *ZNF490, LZTS2, CIZ1, TNK1, PIEZO1, UNC119 and ATOH8*. A gene ontology analysis demonstrated a strong enrichment for regulation of phagocytosis and engulfment, cell maturation, regulation of cell activation as well as of cell proliferation and might therefore be involved in crucial regulatory steps in myeloid differentiation and proliferation (data not shown).

**Fig. 6:**
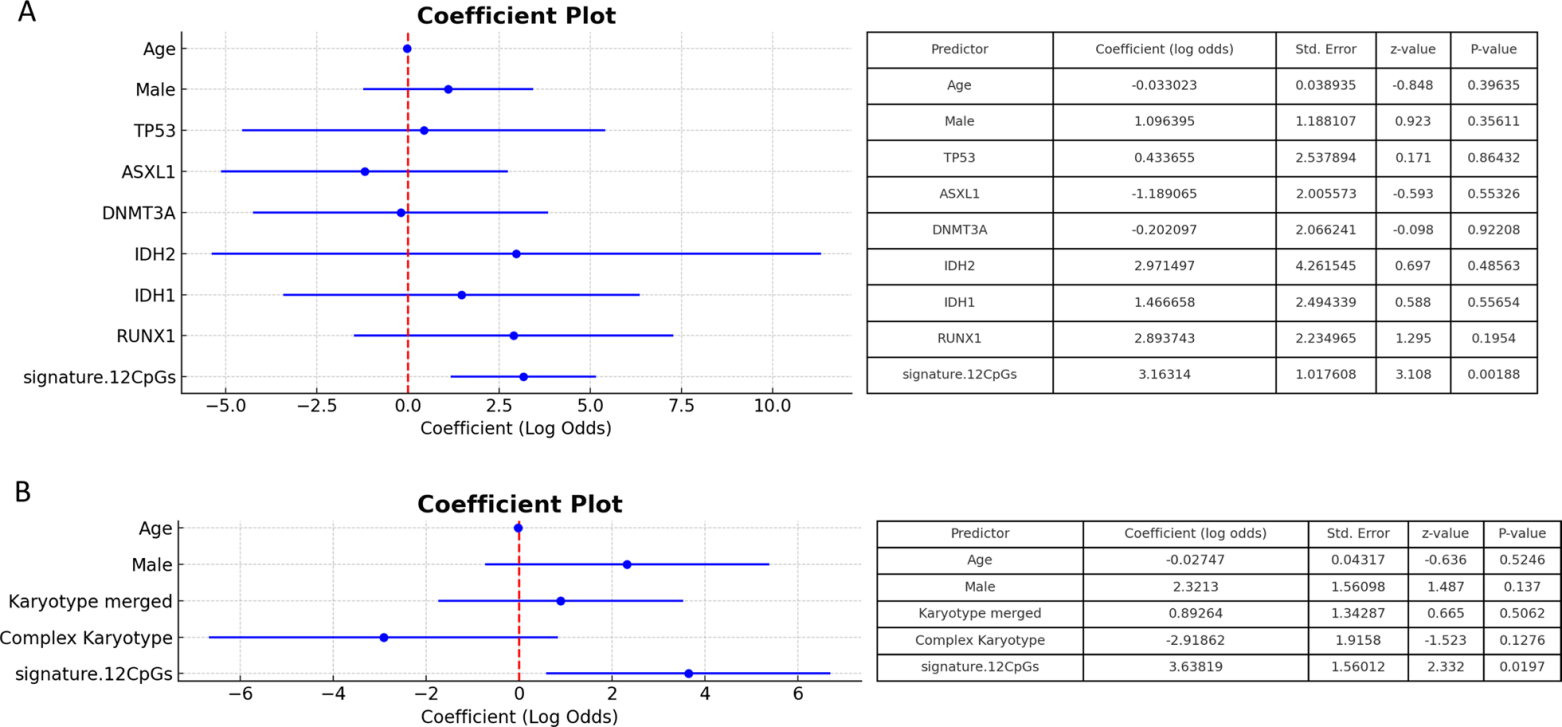
Coefficient plots for multivariable analysis of mutations (**A**) and chromosomal aberrations (**B**). **A** Coefficient plot for contained mutations, age and gender in comparison to the 12-CpG-signature. **B** Coefficient plot for karyotypes, age and gender in comparison to the 12-CpG-signature. Plots include the 95%-confidence interval for each predictor. Values in respective tables are the results from multiple logistic regression modelling. A model containing both mutational and cytogenetic variables could not be fitted because the sample size was too small to estimate all model parameters with sufficient confidence. Due to very small samples sizes for the del(5q)/-5 and t(11q23) groups (*n* = 2 each), both groups were combined with the group „other “ (*n* = 11) for the multivariable analysis. Karyotype merged comprises “del(5q)/-5 “, “t(11q23)“, “other “

### Confirmation of genomewide MCIp-based DMR screening by 450 k infinium human methylation bead chip assay

Validation of the MCIp-derived DMRs was done with HumanMethylation 450k Bead Chip aiming at enabling easy clinical applicability and easy reproducibility of a DNA methylation based predictive signature.

Out of the 50 top hit regions, 80% were represented on the 450 k Bead Chip by at least one CpG probe. In total, remaining top DMRs (*n* = 40) were represented by a total of 105 CpG probes with a variable number of CpG probes per top region (1–7 probes per region) and 25% of regions being defined by a single probe. For *WNT10A*, Fig. 3B illustrates a single CpG-site validation with a correlation of 0.816 for 450 k beta-values of a CpG probe and the reads per kilobase per million mapped reads (RPKM) for the corresponding DMR identified via MCIp-seq. The median correlation coefficient rho over all CpGs was 0.69 (95% confidence interval 0.32–0.87). Additionally, as baseline requirement, a correlation coefficient above the median and unidirectional differences in methylation changes between CR and RD were required for MCIp-Seq and 450 k Bead Chip results to meet the confirmation criteria. Based on the small sample size, the significance level for differential methylation within the 450 k dataset was set at 0.2. With application of these criteria, 90% of DMRs could be quantitatively confirmed via 450 k array-based analysis. 65% of top DMRs met all validation criteria for each CpG probe, 25% met all criteria for at least one CpG probe and 10% of DMRs failed technical confirmation due to insufficient significance levels.

In total, 95 out of 105 CpG probes, contained within 36 out of 40 top hit DMRs, could be confirmed and could subsequently be used to create a multivariable signature for therapy response prediction.

### Generation and refinement of a methylation based predictive classifier based on single distinct CpGs

For an easy and clinically applicable signature, the MCIp-identified regional differences in methylation were aimed to be transformed and compressed into a classifier that contains individual CpGs. A penalized logistic regression model with automated selection of variables was fitted for predicting response to hypomethylating therapy. Logit transformation of 450k data with transition of beta values to M values was performed. Subsequently, fivefold cross validation was done to find optimal penalty parameters as described in the supplement. The resulting classifier comprised 17 CpG dinucleotides which were associated with 12 different genes and two previously undescribed regions (Additional file 2: Fig. 7A). It allowed to perfectly match response or non-response to HMA therapy with AZA when fit to the screening dataset (Additional file 2: Fig. 7B).

### Validation of the DNA methylation based predictive classifier 

Validation of the identified classifier within a validation cohort, derived from the AMLSG 12-09 study group trial cohort (validation cohort, *n* = 97) was performed using MALDI-TOF, a targeted approach for the quantification of DNA methylation at single CpG-site resolution as described earlier [41]. For final data analysis, nine samples were removed from the validation cohort (remaining samples *n* = 88). One sample was removed due to correction of patient response status to early death, another sample was removed due to more than 50% of missing values after generation of mass spectra and the remaining samples were removed due to insufficient amounts of DNA in final quality control before generation of mass spectra.

16 out of 17 classifiers-contained CpG dinucleotides could be addressed with primers suited for mass spectrometry at single CpG-site resolution. Designed primers also encompassed flanking regions with up to 125 bp and included CpGs. The analysis resulted in a total of 152 informative CpG units. After quality control by removal of units with more than 20% of missing values, *n* = 71 informative CpG units remained. For classifier-contained mass spectra 15 out of 17 profiles generated were informative.

When the previously established classifier was mapped to these 15 CpG units as assessed by MALDI-TOF and applied to the validation cohort, validation failed within this cohort. The resulting receiver operating characteristic (ROC) curve was only slightly above the bisecting line and the area under the curve (AUC) was 0,59 resulting in low performance (Additional file 2: Fig. 8).

### Independently validated CpGs, in proximity of the classifier comprised CpGs allow for prediction of therapy response in the validation set (EXP arm), but are not specific for HMA treatment with AZA

We tested, if the signature’s distinction capacity could be preserved with the information from neighboring CpGs by the additionally generated methylation data from flanking regions. Significant differences in methylation were tested for between responders and non-responders by non-parametric Mann–Whitney-U testing both in the EXP and STD arm based on methylation data generated by mass spectrometry. Assessment was performed in the validation cohort and significant differences are visualized in Fig. 4A. There was no overlap with significant hits from the STD arm (Additional file 2: Fig. 9). Significant hits comprised 5 out of 17 target regions from the original classifier.

Based on these results the classifier was recomposed by penalized regression and included 8 out of 15 significantly differentially methylated MassARRAY units, consisting of up to 3 CpGs (Fig. 4B). In total, 12 CpGs were included in the refined classifier. With the refined classifier, prediction of therapy response has an apparent misclassification error of 0.2157, if run without considering subsampling to avoid overfitting. Compared to the original classifier, predictive quality is significantly improved (AUC = 0.924). For the given results, a sensitivity of 93.3% and a specificity of 42.9% can be calculated (Fig. 5A, B; Additional file 2: Fig. 10).

For a final evaluation of classifier quality unbiased from potential overfitting, misclassification error and an AUC were calculated based on 0.632 + bootstrap resampling (Fig. 5C). The refined lasso signature has a bootstrap estimated unbiased misclassification error of about 35%, while the reference error for the null model is about 41%.

In summary, the value of this model for predicting therapy response in new samples is better than the null model. Nevertheless, a substantial error remains. The bootstrap-estimated AUC is about 0.77, which is lower than the AUC computed on the full data set, but better than the AUC for the reference model (Fig. 5C2). Our DNA methylation-based signature which was trained to predict response to therapy was also associated with a trend towards improved OS and a significantly improved EFS (data not shown).

To further assess the classifier’s specificity to HMA treatment it was tested within the STD arm of the validation cohort. With a misclassification error of 0.24 and an AUC of 0.76, the signature unfolds a prediction performance in the STD arm, comparable to the 0.632 + -bootstrap estimates for misclassification error and AUC within the EXP arm (Additional file 2: Fig. 11). Though the recomposed classifier can better discriminate between response and non-response than the null model, it does not reach its genuine goal to discriminate therapy response, specific for AZA.

### Multivariable analysis shows the association of 12-CpG-classifier with treatment response to be independent of potential confounders

Multivariable analysis including potential confounding variables showed that both mutational status of epigenetic modifier genes such as *DNMT3A* and *IDH1/2* and cytogenetics had no impact on the significance of the classifier (Fig. 6A, B). The effect of the 12-CpG signature remained statistically significant in all models, indicating that neither of these variables are important confounders for treatment response in our experimental setting. However, small sample size, the limited panel of mutations and protocol restrictions excluding several recurrent mutations in AML and an overall low fraction of patients with mutations restrict this multivariable analysis.

## Discussion

As no classifier for therapy response prediction to HMA in AML exists, this study aimed at developing a robust, small, cost-effective, and clinically applicable signature for routine testing. This requirement involves fast turnaround times, low amounts of input DNA, as well as moderate technical requirements and manageable costs.

To date, methylation-based biomarkers have not gained acceptance in routine clinical practice, mainly due to limitations in the regions studied, such as promoters, small sample size, or lack of reproducibility in independent cohorts [37, 38]. Of note is a study in 40 patients with chronic myelomonocytic leukemia (CMML) who were treated with DAC [38]. Based on differences in baseline DNA methylation identified via genome-wide next-generation sequencing, a DNA methylation classifier comprising 16 features to distinguish DAC-responders from non-responders was generated and validated in an independent sample set. Prediction accuracy was 87% and decreased to 71% when features were reduced to 6. The authors have suggested that to date, the magnitude of negative results regarding response prediction was largely due to the focus on promoter methylation. Instead, they hypothesized on promoter-distal and intergenic regions as informative for therapy response. Despite this encouraging finding, these results have neither been reproduced nor has an epigenetic classifier in any entity treated with HMA been introduced into clinical routine so far. Recently, a differential methylation signature for response prediction, based on 200 CpG probes, in a set of 75 patients with high risk MDS or sAML was discovered by supervised analysis [37]. Although a promising result, an independent validation cohort was missing. Additionally, within the same set of patients another set of 200 CpG probes was shown to harbor prognostic information but was not independently validated.

Effects of DNMTi-therapy have been shown to include the activation of tumor suppressor genes, the downregulation of oncogenes and the unveiling of an innate antiviral immune response by reactivation of endogenous retroviral pathways respectively retroviruses and inducible, unannotated transcripts, thereby increasing immunogenicity [42–44]. In this context, the likelihood of capturing regions of interest seemed higher by focusing on larger regions (DMRs) instead of single CpGs.

To address these considerable limitations regarding methylation-based analysis, we investigated DNA methylation over the whole genome in an unbiased way. Having chosen a global screening assay (MCIp-seq), we consecutively narrowed down towards a classifier by analysis of regional differences in methylation. Subsequently, by single CpG-site analysis via 450k beadchip assay, we were able to compare discriminatory effects between quantitative evaluation of single CpGs and the CpG-content of defined regions while at the same time increasing the resolution of methylation changes. We consecutively distilled regions of discriminatory power via differential methylation analysis between responding and non-responding patients. Secondly, we evaluated the effect of single-site CpGs within identified regions and included directly neighboring regions allowing for a comparison between the effect of single-site CpGs and regions, irrespective of a region’s CpG density. Based on the generated data, a classifier was trained and subsequently fitted by inclusion of methylation changes of neighboring regions. Without the adjustment of the classifier by this additional information, validation in the test cohort failed.

Among top differentially methylated regions, identified in our screening, was *WNT10A* which is part of the extensively characterized Wnt/ß-catenin signaling pathway and regulates the stability of transcription co-activator ß-catenin [45]. For *MIR3186*, another top DMR, a previous genome-wide differential methylation analysis in salivary gland inflammation in patients with Sjögren’s Syndrome, a chronic, multifaceted autoimmune disease, revealed 57 genes, amongst others *MIR3186,* to be enriched for DMRs in their respective promoters [46]. Leukemia cell lines treated with bortezomib, resulted in upregulation of CCAAT/enhancer binding protein delta (CEBPD) and induced multiple miRNAs such as *MIR3154* amongst others, which were shown to target the 5’-flanking region of *CEBPD* and resulted in epigenetic gene silencing, consistent with a new mechanism in miRNA-mediated gene regulation [47]. *IFT140*, intraflagellar transport protein 140, a subunit of the IFT complex, is essential for retrograde transportation in cilia and mutations as well as dysregulation are linked to syndromic ciliopathies and male fertility [48]. Just recently, a region on chromosome 16, near *IFT140*, has been described as differentially methylated and associated with pancreatic cancer risk in an epigenome-wide association study [49]. Moreover, *IFT140* has been shown to be differentially methylated in fetal alcohol spectrum disorder [50]. Finally, for *IGF2BP1*, the oncofetal IGF2 mRNA binding proteins (IGFBPs) are upregulated in various cancer entities and have been shown to possess a distinct conservation of highly oncogenic potential throughout a panel of five cancer-derived cell lines [51]. Together with data from knockout mouse models, *IGF2BP1* seems to enhance an aggressive tumor cell phenotype by antagonizing miRNA-impaired gene expression [51].

Taken together, the DMRs, distilled from our analysis, have been shown to be not only differentially methylated in other entities as well, but to be also involved in key regulatory processes associated with oncogenic transformation as well as with defining distinct phenotypic disease characteristics. In addition, this study confirmed previous findings, such as the dominance of distal regulatory elements among response associated DMRs [38]. This supports the idea of relevant DMRs not being evenly distributed across the genome, but instead being enriched at distinct regions and is in line with recent reports of aberrant gene expression being correlated with aberrant DNA methylation, e.g. at enhancers in cell lines from various entities [52].

To confirm our findings, we consecutively evaluated our classifier in a validation set of patients from the same clinical trial. While identification of responders with our signature was possible with reasonable discriminative power (AUC = 0.77), response prediction was also possible within the STD treatment group of the validation set, resulting in a nearly identical AUC (0.76). This result highlights the predictive power of our signature, but at the same time illustrates that it was not possible for us to identify a HMA treatment specific response prediction. One major reason for this outcome might be the design of the AMLSG 12-09 trial. The rationale for incorporating AZA in AMLSG 12-09 was based on its hypomethylating properties when administered at a low dose rather than its cytotoxic effects observed at higher doses in a patient cohort, ineligible for targeted therapy [53]. As the trial failed to support the substitution of AraC by AZA in intensive induction therapy, it might probably not be entirely possible to detect an HMA-specific signature in a patient cohort where a strong chemotherapy backbone is part of both trial arms. Both event-free and overall survival were significantly inferior in the AZA containing arms as compared to the standard therapy arm resulting in a negative trial. Thus, the identified signature might need to be applied to other data sets to evaluate its discriminatory power.

It is possible that the data used to train our classifier are not sufficiently representative in terms of patient numbers and the distribution of patient (genetic) characteristics to build a robust classifier and to demonstrate statistical independence in multivariable testing. The limitation of the small sample size results from the limited availability of samples from the AMLSG 12-09 trial which is due to the study design. Despite the limited number of patients, the fact that the generation of our predictive epigenetic signature was based on a prospective randomized trial represents a key quality feature. Therefore, we are confident that our approach in terms of analysis strategy, potential limitations and pitfalls is a valuable contribution for the development and evaluation of predictive biomarkers for hypomethylating agents.

Second, the bone marrow blast count was the only significant difference in an otherwise homogeneous sample cohort with a significantly higher bone marrow blast count in the EXP arm. This fact has the potential to introduce a bias into the differential methylation analysis by affecting the alignment of identified DMRs between the EXP and STD arms such that correction for unique DMRs in the EXP arm could be ineffective. This might lead to a higher risk of identifying DMRs which account for rather global, chemotherapy-associated effects. Regarding the different frequency of *DNMT3A* mutations in the cohorts, we consider a bias in the construction of our classifier unlikely, because no association with genotypes was found in the multivariable analysis. In addition, *DNMT3A* or further DMRs near *DNMT3A* were not part of the DMR top list and were therefore not included in the final classifier.

Third, the use of different platforms for DNA methylation assessment could have the potential to introduce error and variability into the analyses. In this case, the overall goal was to develop a small, robust, and easily applicable predictive signature starting from genome-wide unbiased screening. This goal required the sequential application of different techniques with different characteristics. The use of different assays allowed us to highlight the informational value of individual CpG units compared to regions in terms of differential methylation. Nevertheless, we cannot exclude that technical aspects may have contributed to the lack of discriminative power for the HMA response signature by this approach. In conclusion, we were able to show that the methylation status of regions, as determined by MCIp-seq, can be confirmed via quantitative analysis of representative CpG units. The identified regions might even be functionally linked. our technical approach with confirmed CpG units showed a loss in discriminatory power that could be compensated for by inclusion of close-by CpG units resulting in a predictive classifier. While assessment of the classifier within the STD arm confirmed response prediction, it was not HMA specific. Thus, our findings suggest that a predictive epigenotype seems to be carrying its information on methylation on a complex, genome wide scale and is confined to regions, rather than to single CpGs. Trials with a larger sample cohort and a more representative cross-section of the otherwise heterogeneous AML biology are needed to pin down subsets of AML patients, for whom a predictive tool set might be developable.

In summary this work once again demonstrates that there seems to be no easy way to determine prediction to HMA agents based on pretreatment methylation parameters. As has been proposed, response prediction in HMA, Venetoclax or other therapies might only be harnessed when longitudinally monitoring the methylation status of patients treated with HMA [30]. In the context of increasing therapeutic complexity with combination regimens including HMA, response prediction might even become more complicated. Further studies are needed to evaluate the dynamics of methylation changes over the course of treatment and to correlate them with therapy response. In this scenario, a predictive classifier must be significantly faster in predicting response than the natural course of the disease.

## Conclusions

This study aimed at developing a fast and affordable predictive classifier for therapy response prediction to HMAs in AML. While previous attempts at utilizing methylation-based biomarkers have shown promise, none have been consistently reproduced or introduced into clinical practice. Here, for the first time, we investigated DNA methylation profiles with a genome-wide screening that is not limited to specific genomic regions which we consider to be a prerequisite for the successful development of epigenetic predictive signatures.

While the identified classifier can predict response, it is not specific to HMAs, suggesting that the methylation information is complex and genome-wide, confined to regions rather than single CpG sites. Based on our data, it is unlikely that a response prediction can be derived from a simple signature containing only a few CpG dinucleotides.

A potential signature is likely to be highly dependent on the therapeutic context, e.g., the HMA combination partners as in our case, where the chemotherapy backbone could be dominant and mask the identification of an HMA-specific signature.

In summary, our analyses are a step towards the development of epigenetic biomarkers and highlight potential problems and relevant aspects that should be considered future development of predictive epigenetic signatures.

## Patients and methods

### AMLSG 12-09 trial

All samples were obtained from the AMLSG 12-09 trial (ClinicalTrials.gov number: NCT01180322, EudraCT number: 2009-016142-44), a prospective, randomized, multicenter, controlled four-armed phase-II design [39]. This trial tested the rationale of substituting cytarabine (araC) in the standard arm (STD) by different schedules of azacytidine (experimental arm, EXP) in idarubicin and etoposide containing induction therapy of newly diagnosed AML patients. 277 adult patients (age range 18–82) were enrolled between October 2010 and March 2012. In this trial, molecularly defined subtypes allowing for genotype specific therapy approaches such as patients with mutated *NPM1*, AML with *FLT3*-ITD, *PML-RARA* fusion, and CBF-AML were excluded. Induction therapy was followed by maintenance therapy with 5-azacitidine for two years. Details of the trial design and analysis are given in the final trial report [42]. In the final analysis, regarding the primary endpoint of therapy response, the substitution of cytarabine by azacytidine failed to improve response rates [39]. All study arms were associated with a worse outcome than the standard arm.

### Patients and bone marrow samples

Mononuclear cells from pretreatment bone marrow aspirates were available from *n* = 155 patients following patients’ informed consent under the institutional review of ethics-committee of Ulm University (number: 175/10, October 11, 2010). Informed consent was obtained in accordance with the Declaration of Helsinki and approval was obtained from institutional review committees at participating centers.

A screening set was assembled from 58 samples with 18 patients receiving standard (STD) therapy (Ida/AraC/Eto) and 40 patients receiving experimental (EXP) treatment (Ida/Aza/Eto) within the AMLSG 12-09 trial. For validation of differentially methylated regions, an independent subset of 97 patient samples derived from the AMLSG 12-09 collective was obtained. The validation cohort consisted of 40 patients receiving standard therapy and 57 patients receiving experimental treatment. In accordance with standard ELN criteria, responders were defined by achieving complete response (CR) defined as < 5% bone marrow blasts, an absolute neutrophil count ≥ 1,0 G/L, a platelet count of > 100 G/L, no blasts in the peripheral blood and no extramedullary leukemia.

### DNA extraction and bisulfite conversion

DNA from bone marrow mononuclear cells of AML patients was isolated using the QIAmp DNA Mini Kit (QIAGEN) according to the manufacturer’s instructions.

Bisulfite conversion of genomic DNA was performed with EZ DNA Methylation™ kit from Zymo Research (Zymo Research, Irvine, USA) according to the manufacturer’s protocol using 500 ng of genomic DNA per sample. Conversion rate of bisulfite treatment was tested with PCR amplification of the *SALL3* gene locus as described previously [43].

### Genome-wide DNA methylation screening by methyl-CpG immunoprecipitation (MCIp)-seq

Methyl-CpG immunoprecipitation (MCIp) was performed as described previously [44]. In brief, a total of 3.0 μg DNA from bone marrow mononuclear cells was sonicated with the Covaris S220 focused-ultrasonicator (Covaris, Woburn, USA) to fragments of an optimal fragment size ranging between 100 and 200 bp as monitored via capillary electrophoresis on an Agilent 2100 Bioanalyzer (Agilent Technologies, Santa Clara, USA). Sonicated DNA was enriched with 90 μg of purified methyl-CpG-binding domain-Fc protein coupled to 60 μl protein A-coated magnetic beads. Enrichment resulted in increased mean fragment size of about 40 bp. Subsequently, DNA was eluted by incubation with increasing salt concentrations (fraction A, 300 mM; B, 400 mM; D, 550 mM; F, 1000 mM). Non-methylated alleles elute at low-salt while methylated alleles elute at high-salt concentration. Desalted eluates were controlled for enrichment of methylated DNA by real-time PCR via quantification of abundance of the housekeeping gene GAPDH and a selected ribosomal RNA gene promoter with variable expression [1]. Enriched fragments were subsequently sequenced on the Illumina HiSeq™ 2000 platform as described earlier [45]. Details are to be found in the supplement. MCIp-enriched methylated DNA fragments were submitted to the DKFZ Genomics and Proteomics Core Facility for library preparation and next-generation sequencing. Afterwards, fragmented DNA was end-repaired and ligated to Illumina-paired end adaptors using NEBnext DNA Library Prep Master Mix Set (New England Biolabs) in accordance with the manufacturer’s instructions. Adapter ligated libraries were directly amplified by 14 cycles of PCR with the standard Illumina index primers and distributions were validated using the Agilent Bio- analyzer before it was quantified by a Qubit fluorometer (Invitrogen). The libraries were sequenced on the Illumina HiSeq 2000 sequencer (50 bp, single read 50 bp) using standard Illumina protocols. Details about MCIp-seq and bioinformatic analyses are given in Additional file 1.

### Quality assessment, bioinformatic processing and data analysis of MCIp-seq raw data

Sequencing reads were aligned to the hg19 genome assembly of the human reference genome using the Burrows-Wheeler Alignment tool. Aligned reads were further processed by merging lane-level data and removing duplicates. The remaining uniquely mapped reads were converted to Sequence Alignment Map or Binary Alignment Map formats using SAMtools. Read counts of each sample were normalized for total read length and the number of sequencing reads (reads per kilobase per million mapped reads; RPKMs). Peak calling was per- formed using the software HOMER (v4.4).

### Array-based quantitative assessment of DNA methylation applying the Infinium® Human Methylation 450k Bead Chip from llumina®

Quantitative DNA methylation assessment was performed with the Infinium® HumanMethylation 450k Bead Chip for comprehensive genome-wide coverage of methylation data as described previously [38]. Logit transformation of 450k data with transition of beta values to M values was performed. Quality control of generated data was performed with the RnBeads package for R [46]. For background correction, the NOOB method [47] and for data normalization the BMIQ algorithm [48] were applied.

### Quantitative assessment of DNA methylation applying MassARRAY® technology from Sequenom®

Quantitative DNA methylation analysis was performed using MALDI-TOF mass spectrometry (MassARRAY, Sequenom, San Diego, USA) as previously described [41]. Target regions for DNA methylation analysis were designed to yield maximum information for single CpG dinucleotides by in silico processing using custom R-based scripts. Primers were designed, tested, and optimized for PCR amplification. *In-silico* bisulfite conversion, *in-silico* fragmentation, and fragment yield estimation in mass spectrometry using the RSeqMeth package were considered in primer design. [49]. Final primer pairs were fitted for a fragment length between 200 and 500 bp, an ideal primer length of 22–25 bp, an ideal annealing temperature of 60 °C, a maximum tolerated difference in annealing temperature between forward and reverse strand primers of 5 °C, low overall thymine content, cytosine-rich 3’end content and obligate exclusion of CpG dinucleotides. A ratio of informative to total CpGs of at least 0.7 was met. Target gene regions were amplified by PCR after sodium-bisulfite modification of genomic DNA. Subsequently deoxynucleotides in the PCR reaction were inactivated by dephosphorylation using shrimp alkaline phosphatase (SAP). By tagging the reverse PCR primer with the T7 recognitions sequence, a single-stranded RNA copy of the template was generated by in vitro transcription. After base specific (U-specific) cleavage by RNase A, the cleavage products were then analyzed using MALDI-TOF mass spectrometry. Cleavage product signals with a 16 Da shift (or a multiple thereof) are representative for methylation events and signal intensity is correlated with the degree of DNA methylation. For quantitative methylation assessment within the validation cohort, out of 152 informative CpG units, all units with more than 20% of missing values were excluded with *n* = 71 informative distinct CpG units remaining. Remaining missing values were computed by single imputation using k-nearest neighbor imputation [50]. For CpG probes assessed by several CpG units in mass spectrometry, mean values across CpG units were calculated.

### Analytical strategy and statistical analysis

Details on the analytical strategy and statistical analysis are found in the supplement. In brief, based on the February 2009 assembly of the human genome, DMRs were identified based on a genome binning approach by grouping the genome into factions of 500 bp length. Reads were assigned and normalized to each 500 bp window with the HOMER software. Uninformative regions were filtered. Bins with no reads across all or across all but one sample were discarded. Differential methylation was calculated with edgeR [51]. Top lists of DMRs were generated for STD and EXP-arms respectively and ranked according to effect size and *p*-values. Overlaps between both lists were excluded and a top list was generated. Quantitative methylation analysis was performed via HumanMethlyation 450k Bead Chip. Via penalized logistic regression analysis and fivefold cross-validation for identification of optimal penalty parameters a predictive classifier was trained and assessed with a test cohort.

### Supplementary Information

**Additional file 1.** Patients & Methods. This section provides extended details on the handling of patient samples and specifics about DNA extraction and bisulfite conversion as well as genome-wide DNA methylation screening by methyl-CpG immunoprecipitation (MCIp)-seq. Moreover a detailed description of data and sample processing for the Array-based quantitative assessment of DNA methylation (Infinium® Human Methylation450 Bead Chip, lllumina®) and the quantitative assessment of DNA methylation (MassARRAY® technology, Sequenom®) are provided. The section also contains details on the analytical strategy and statistical analyses.

**Additional file 2. Fig S1.** Distribution of somatic mutations and cytogenetics in the screening cohort. Overview of somatic mutations in epigenetic modifier genes. No association between mutations in epigenetic modifiers and therapy response as well as methylation patterns was observed. The overall screening sample set did not exhibit distinct clustering patterns. **Fig S2.** Unsupervised hierarchical clustering of the 1.000 (**A**) and 10.000 (**B**) most variable regions Assessment of the impact of karyotypes and mutations in epigenetic modifier genes by unsupervised hierarchical clustering of the top 1000 and top 10000 most variable CpG regions in the screening cohort. **Fig S3.1–3.10.** Distribution of mutations in epigenetic modifier genes and cytogenetic aberrations in the top 10 DMRs.The distribution of mutations in the epigenetic modifier genes as well as the distribution of cytogenetic aberrations in the top 10 DMRs (*WNT10A, ZNF490, LZTS2, CIZ1, TNK1, LOC100133991, PIEZO1, C5orf65, UNC119, ATOH8*) between responding and refractory patients is shown. There is no segregation of mutation patterns with response in the selection of DMR candidates. **Fig S4.** Principal Component Analysis (PCA) on 500-bp bins after primary filtering of uninformative regions on all samples (**A**) and on the EXP arm (**B**) within the screening cohort. Principal component analysis based on 664,227 bins for the overall sample set and the experimental therapy arm. Labeled samples indicate extreme values in read count numbers, i.e. the top and bottom 5% read count values. Blue and red dots represent data points that were identified as potential outliers based on either extremely low total read counts, as shown in blue, or extremely high total read counts, as shown in red. For subsequent steps of differential methylation analysis, blue samples, i.e. unsaturated samples with low total read counts were ignored. **Fig S5.** Distribution of differentially methylated regions (DMRs) across the genome with (**A**) a Box-Whiskers plot indicating distribution of DMRs within the total set of DMRs filtered for bins with positive read counts across all samples and with (**B**) bar plots indicating the distribution of genomic annotations in the set of top candidates. **Fig S6.** GC content distribution and the relationship between GC content and transcriptional start sites (TSS) with (**A**) regions with higher GC content showing an over-representation in the set of top candidates, irrespective of data normalization with CQN and (**B**) regions in the set of top candidates showing a close relation to transcriptional start sites. “Top hits (std)” denominate data not normalized by application of CQN. A graph for CQN normalized data is included for (**A**) and (**B**). **Fig S7.** Components of the primary classifier with (**A**) a multivariable signature for therapy response prediction containing 17 probes. CpG dinucleotides are associated with 12 genes and two previously undescribed regions and (**B**) a prediction matrix for therapy response (CR - green color) generated by applying a penalized logistic regression model (“elastic-net penalty”) to the 450k M-values within the set of validated candidates. The y-axis gives the probability for refractory disease (RD - red color). **Fig S8.** ROC curve for the primary 450k elastic net signature linear predictor. Receiver operating characteristic curve for the 17 CpG containing classifier as assessed within the validation cohort. Both sensitivity and specificity do not allow for a reliable prediction of therapy response.**Fig S9.** Significantly differentially methylated flanking CpGs as assed by MassARRAY for classifier refinement showing significant DMRs in EXP arm only. **A** and **B** show Manhattan plots for univariable testing of candidate regions based on MassARRAY data from validation sample set. Significant hits are limited to patients treated with a combination therapy regimen as shown on the left whereas the group of patients receiving standard therapy showed no significant hits. **Fig S10.** Probability estimates and misclassifications for the refined classifier. Probability estimates for complete response to demethylating therapy. CR and RD indicate complete response and refractory disease, respectively. Unstained dots indicate samples with correct predictions to therapy, whereas red dots indicate misclassifications. **Fig S11.** ROC curve for the lasso signature linear predictor applied to STD arm. Evaluation for 5-azacytidine treatment arm specificity by application of the classifier onto the STD arm results in a misclassification error of 0.24 and an AUC of 0.76. The result is comparable to the .632+-bootstrap estimates for the misclassification error and the AUC for the EXP arm. This finding indicates unspecificity in the EXP arm.

**Additional file 3.** Overview of predictive epigenetic biomarkers. The table provides a short review of published predictive biomarkers related to DNA-methylation and hypomethylating agents.

**Additional file 4.** Patient characteristics within the screening cohort both for the standard arm as well as for the experimental arm are provided.

**Additional file 5.** Overview of top differentially methylated regions within the experimental treatment arm. Selected candidate regions are marked in green. Logarithmic fold change (logFC) and p-values are given for all regions. Respective values form the standard arm are highlighted in yellow. All regions are arranged in ascending order of p-values (experimental arm).

## Data Availability

The datasets used and/or analysed during the current study are available from the corresponding author on reasonable request.
