# Supplementary material for: Predictive value of DNA methylation patterns in AML patients treated with an azacytidine containing induction regimen
Source: Clin Epigenetics. 2023 Oct 26;15:171. doi: 10.1186/s13148-023-01580-z (PMC10601277; doi:10.1186/s13148-023-01580-z)
Supplement: Supplementary file 1 — Additional file 1. Patients & Methods. This section provides extended details on the handling of patient samples and specifics about DNA extraction and bisulfite conversion as well as genome-wide DNA methylation screening by methyl-CpG immunoprecipitation (MCIp)-seq. Moreover a detailed description of data and sample processing for the Array-based quantitative assessment of DNA methylation (Infinium® Human Methylation450 Bead Chip, lllumina®) and the quantitative assessment of DNA methylation (MassARRAY® technology, Sequenom®) are provided. The section also contains details on the analytical strategy and statistical analyses. [file 13148_2023_1580_MOESM1_ESM.docx]

**Predictive value of DNA methylation patterns in AML patients treated with an azacytidine containing induction regimen.**

Maximilian Schmutz^1,2^, Manuela Zucknick^2,3^, Richard Schlenk^4,5^, Daniel Mertens^6,7^, Axel Benner^8^, Dieter Weichenhan^2^, Oliver Mücke^2^, Konstanze Döhner^9^, Christoph Plass^2^, Lars Bullinger^10.11^, Rainer Claus^1,2,12$^

^1^Hematology and Oncology, Medical Faculty, University of Augsburg Medical Center, Augsburg, Germany.

^2^ Division Epigenomics and Cancer Risk Factors, German Cancer Research Center (DKFZ), Heidelberg, Germany

^3^ Oslo Centre for Biostatistics and Epidemiology, University of Oslo, Oslo, Norway.

^4^ National Center of Tumor Diseases, NCT-Trial Center, German Cancer Research Center and Heidelberg University Hospital, Heidelberg, Germany.

^5^ Department of Internal Medicine V, Heidelberg University Hospital, Heidelberg, Germany.

^6^ Cooperation Unit "Mechanisms of Leukemogenesis", German Cancer Research Center, Heidelberg, Germany.

^7^ Division of Chronic Lymphocytic Leukemia, Department of Internal Medicine III, Ulm University Medical Center, Ulm, Germany.

^8^ Division of Biostatistics, German Cancer Research Center (DKFZ), Heidelberg, Germany.

9 Department of Internal Medicine III, University Hospital of Ulm, Ulm, Germany

10 Berlin Institute of Health at Charité-Universitätsmedizin Berlin, Germany.

11 Department of Hematology, Oncology, and Tumorimmunology, Campus Virchow Klinikum, Berlin, Charité-Universitätsmedizin Berlin, Corporate Member of Freie Universität Berlin and Humboldt-Universität zu Berlin, Berlin, Germany.

12 Pathology, Medical Faculty, University of Augsburg Medical Center, Augsburg, Germany.

# Supplement

# Methods *Genome-wide DNA methylation screening by methyl-CpG immunoprecipitation (MCIp)-seq*

Sonicated DNA was enriched with 90 μg of purified methyl-CpG-binding domain-Fc protein coupled to 60 μl protein A-coated magnetic beads. Enrichment resulted in increased mean fragment size of about 40 bp. Subsequently, DNA was eluted by incubation with increasing salt concentrations (fraction A, 300 mM; B, 400 mM; D, 550 mM; F, 1000 mM). Non-methylated alleles elute at low-salt while methylated alleles elute at high-salt concentration. Desalted eluates were controlled for enrichment of methylated DNA by real-time PCR via quantification of abundance of the housekeeping gene *GAPDH* and a selected ribosomal RNA gene promoter with variable expression [1].

Highly enriched (methylated) fragments were sequenced on the Illumina HiSeq™ 2000 platform as described earlier [2]. Read length was 50 bp single-end. For methylome characterization, a total sequencing depth of 20 million reads per flow cell were determined to achieve sufficient coverage.

Conditional Quantile Normalization

As the MCIp assay preferentially enriches CpG dense regions, thereby decreasing the detection probability of regions with lower GC content but higher methylation levels, normalization modelling with CQN (conditional quantile normalization) was tested and showed no impact on the generated results (**Supplemental Figure 4**). Conditional quantile normalization, as introduced by Hansen et al., is an algorithm which unifies a regression model and moreover offers the advantage of dealing with systematic bias such as GC content [3]. CQN exerts power in dealing and eliminating fold change and GC content dependence in sequencing data therefore allowing to reduce variability and bias when working with data generated from sequencing.

*Bioinformatic processing of raw data and the genome binning approach*

Raw sequencing data was obtained in fastq file format. Quality assessment of data was performed with FastQC [4]. Saturation coefficients of sequenced samples were established as primary quality parameter to address the coverage profile. Mapping of raw data was performed using the Burrows-Wheeler Transformation [5] [6]. Raw read alignment was based on the February 2009 assembly of the human genome (hg19, GRCh 37 Genome Reference Consortium Human Reference 37 (GCA_000001405.1)). Generated reads were sorted, barcodes, duplicates and bad quality reads were removed. For downstream analyses, alignment data was transformed into BED file format. Assignment of read counts was performed with the open-source HOMER software for motif discovery and next-generation sequencing analysis [7]. Further downstream processing and spreadsheet analysis was performed using the R language environment for statistical computing [8]. All R scripts are available on demand.

Based on the February 2009 assembly of the human genome, DMRs were identified based on a genome binning approach. To evaluate the effect of both regions and single CpGs on the prediction of therapy response on a whole genome level, the human genome was grouped into 6,191,368 fractions of 500 bp length and subsequently read counts of corresponding bins were calculated and normalized with the HOMER software. Uninformative regions were filtered and bins with no reads across all or across all but one sample were discarded. For remaining regions, differential methylation was tested for with the edgeR package at a false discovery rate of 5% [9] [10]. Top lists of DMRs were generated for STD and EXP-arms respectively and ranked according to effect size and p-values. Overlaps between both lists were excluded to exclude potential non-HMA related effects on methylation. 40 top hits were chosen for further work up.

*Statistical analysis*

Based on the February 2009 assembly of the human genome, DMRs were identified based on a genome binning approach.

EdgeR-based assessment of differential methylation was based on nonparametric, double-sided Wilcoxon rank-sum testing, as described earlier. [11] The method is based on the negative binomial distribution. It is designed for the analysis of read counts from various gene expression experiments which are primarily based on RNA-Seq technologies and allows for a sharp separation between technical and biological variation. False discovery rate (FDR) was determined according to the method of Benjamini-Hochberg [12]. DMRs with a p-value ≥ 0,05 were excluded.

Technical validation of target regions identified via MCIp-Seq was based on quantitative methylation assessment via HumanMethylation450k Bead Chip as described above. Multiple criteria for sufficient validation, all to be met for individual DMRs, were chosen. A strong and distinct correlation between 450k beta-values of CpG probes and reads per kilobase per million mapped reads (RPKM) within corresponding bins, assessed on a computed log2 scale, calculated p-values for differences in beta regression levels between responding and non-responding samples, required to be below 0.2 based on the small number of sample size and lack of sample size estimation, and congruency of methylation changes between responding and non-responding patients both in MCIp-Seq- and 450k-generated data were set as requirement.

Due to a highly dimensional input space, for generation of a predictive gene set, penalized regression was used in order to calculate a misclassification error in dependence of an elastic net penalty term [13].

Based on the assumption that a small number of features accounts for a particular effect, standard linear regression was amended by penalty parameters thus allowing for a low signal-to-noise ratio and reduction of over-fitting for generating a sparse model. This results in a maximum likelihood estimate.

Logit transformation was computed to modify HumanMethylation450 Bead Chip data prior to penalized logistic regression analysis. Outputted Beta-values are characterized by heteroscedasticity, especially in the context of CpG sites with extreme methylation values and expected high levels of variance throughout the dataset. Logit transformation allows for attachment of equal variance to all proportions. A logit L is defined as the logarithm of odds, i.e. a probability p divided by its complementary probability 1-p. As a result, the dependent variable is not limited to an interval between 0 and 1 but can instead take any value between positive and negative infinite values thus allowing for an approximation towards normal distribution.

For detection of optimal penalty parameters, cross-validation was utilized. Cross-validation involves a multi-level approach with a training and a previously unknown test set. Generally exhaustive and non-exhaustive approaches can be distinguished and several distinct approaches such as simple cross-validation, stratified cross-validation or leave-one-out-cross-validation can be applied. The process involves splitting the known dataset into at least two fractions, one for feature extraction and one for subsequent model testing. For this study, leave-one-out-cross-validation, as special case of leave-p-out-cross validation, was applied. An iterated procedure of splitting the dataset for feature extraction and assessment of model validity was performed by choosing p observations and assessing predictive validity in the remaining observations until all possible splits for a given dataset are assessed.

For classifier refinement, a penalized likelihood regression model was utilized: A lasso model was generated to induce sparse solutions with a small number of variables.

**References**

1. Schlesinger, S., et al., *Allelic inactivation of rDNA loci.* Genes Dev, 2009. **23**(20): p. 2437-47.

2. Mardis, E.R., *Next-generation DNA sequencing methods.* Annu Rev Genomics Hum Genet, 2008. **9**: p. 387-402.

3. Hansen, K.D., R.A. Irizarry, and Z. Wu, *Removing technical variability in RNA-seq data using conditional quantile normalization.* Biostatistics, 2012. **13**(2): p. 204-16.

4. Kroll, K.W., et al., *Quality Control for RNA-Seq (QuaCRS): An Integrated Quality Control Pipeline.* Cancer Inform, 2014. **13**(Suppl 3): p. 7-14.

5. Li, R., et al., *SOAP2: an improved ultrafast tool for short read alignment.* Bioinformatics, 2009. **25**(15): p. 1966-7.

6. Li, H. and R. Durbin, *Fast and accurate long-read alignment with Burrows-Wheeler transform.* Bioinformatics, 2010. **26**(5): p. 589-95.

7. Fehniger, T.A., et al., *Next-generation sequencing identifies the natural killer cell microRNA transcriptome.* Genome Res, 2010. **20**(11): p. 1590-604.

8. Team, R.D.C., *R: A language and environment for*

*statistical computing. R Foundation for Statistical Computing,*

*Vienna, Austria.* 2008.

9. Robinson, M.D. and G.K. Smyth, *Small-sample estimation of negative binomial dispersion, with applications to SAGE data.* Biostatistics, 2008. **9**(2): p. 321-32.

10. Robinson, M.D., D.J. McCarthy, and G.K. Smyth, *edgeR: a Bioconductor package for differential expression analysis of digital gene expression data.* Bioinformatics, 2010. **26**(1): p. 139-40.

11. McCarthy, D.J., Y. Chen, and G.K. Smyth, *Differential expression analysis of multifactor RNA-Seq experiments with respect to biological variation.* Nucleic Acids Res, 2012. **40**(10): p. 4288-97.

12. Hochberg, Y.B.a.Y., *Controlling the False Discovery Rate: A Practical and Powerful Approach to Multiple Testing.* Journal of the Royal Statistical Society. Series B (Methodological), 1995. **57**(1): p. 289-300.

13. Zou H, H.T., *Regularization and variable selection via the elastic net.* JR Statist SoJ, 2005: p. 301-320.
