# Supplementary material for: Predictive value of DNA methylation patterns in AML patients treated with an azacytidine containing induction regimen
Source: Clin Epigenetics. 2023 Oct 26;15:171. doi: 10.1186/s13148-023-01580-z (PMC10601277; doi:10.1186/s13148-023-01580-z)
Supplement: Supplementary file 2 — Additional file 2. Fig S1. Distribution of somatic mutations and cytogenetics in the screening cohort. Overview of somatic mutations in epigenetic modifier genes. No association between mutations in epigenetic modifiers and therapy response as well as methylation patterns was observed. The overall screening sample set did not exhibit distinct clustering patterns. Fig S2. Unsupervised hierarchical clustering of the 1.000 (A) and 10.000 (B) most variable regions Assessment of the impact of karyotypes and mutations in epigenetic modifier genes by unsupervised hierarchical clustering of the top 1000 and top 10000 most variable CpG regions in the screening cohort. Fig S3.1–3.10. Distribution of mutations in epigenetic modifier genes and cytogenetic aberrations in the top 10 DMRs.The distribution of mutations in the epigenetic modifier genes as well as the distribution of cytogenetic aberrations in the top 10 DMRs (WNT10A, ZNF490, LZTS2, CIZ1, TNK1, LOC100133991, PIEZO1, C5orf65, UNC119, ATOH8) between responding and refractory patients is shown. There is no segregation of mutation patterns with response in the selection of DMR candidates. Fig S4. Principal Component Analysis (PCA) on 500-bp bins after primary filtering of uninformative regions on all samples (A) and on the EXP arm (B) within the screening cohort. Principal component analysis based on 664,227 bins for the overall sample set and the experimental therapy arm. Labeled samples indicate extreme values in read count numbers, i.e. the top and bottom 5% read count values. Blue and red dots represent data points that were identified as potential outliers based on either extremely low total read counts, as shown in blue, or extremely high total read counts, as shown in red. For subsequent steps of differential methylation analysis, blue samples, i.e. unsaturated samples with low total read counts were ignored. Fig S5. Distribution of differentially methylated regions (DMRs) across the genome with (A) a Box-Whiskers [file 13148_2023_1580_MOESM2_ESM.pptx]

## Slide 1
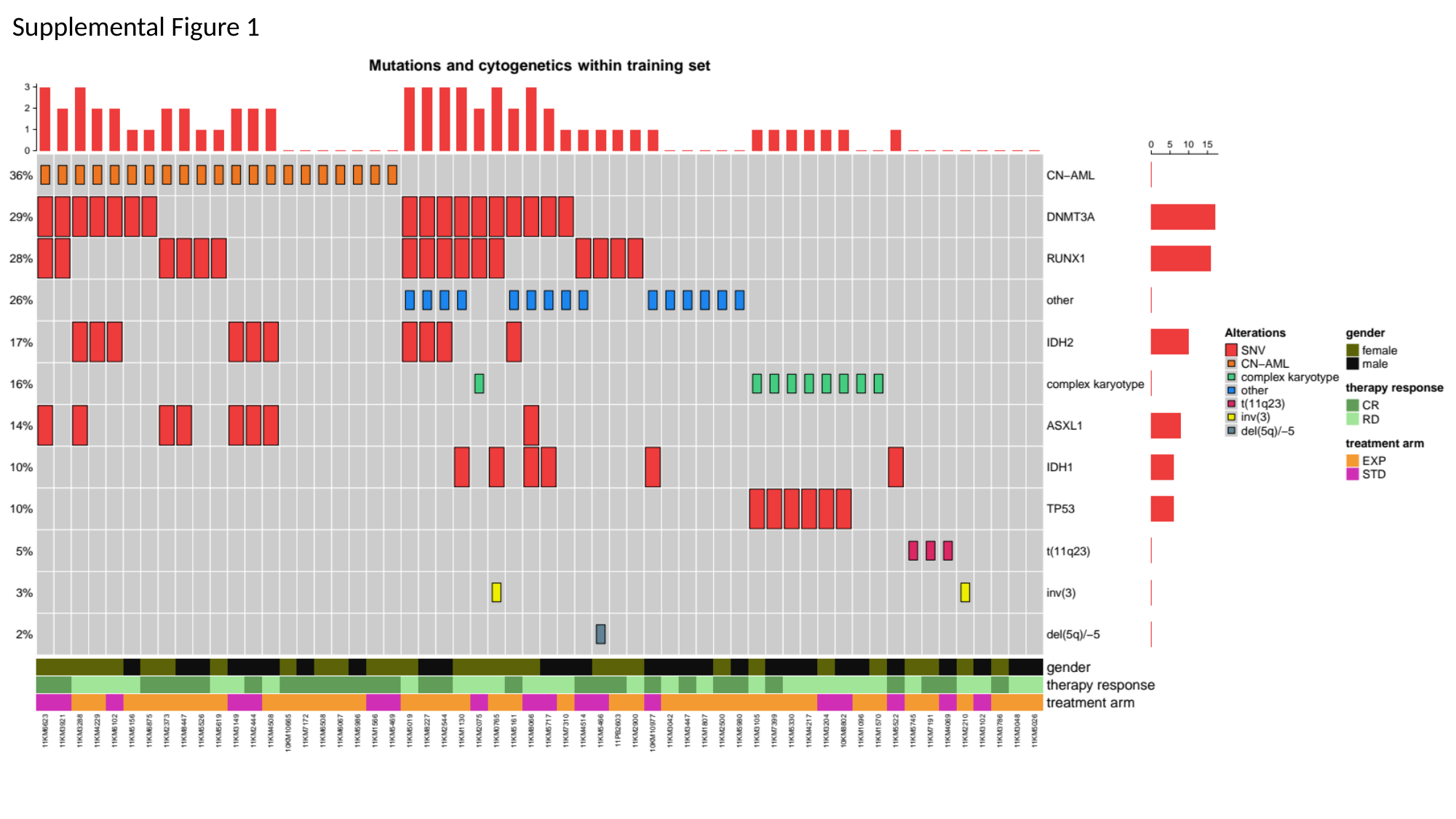

Supplemental Figure 1

## Slide 2
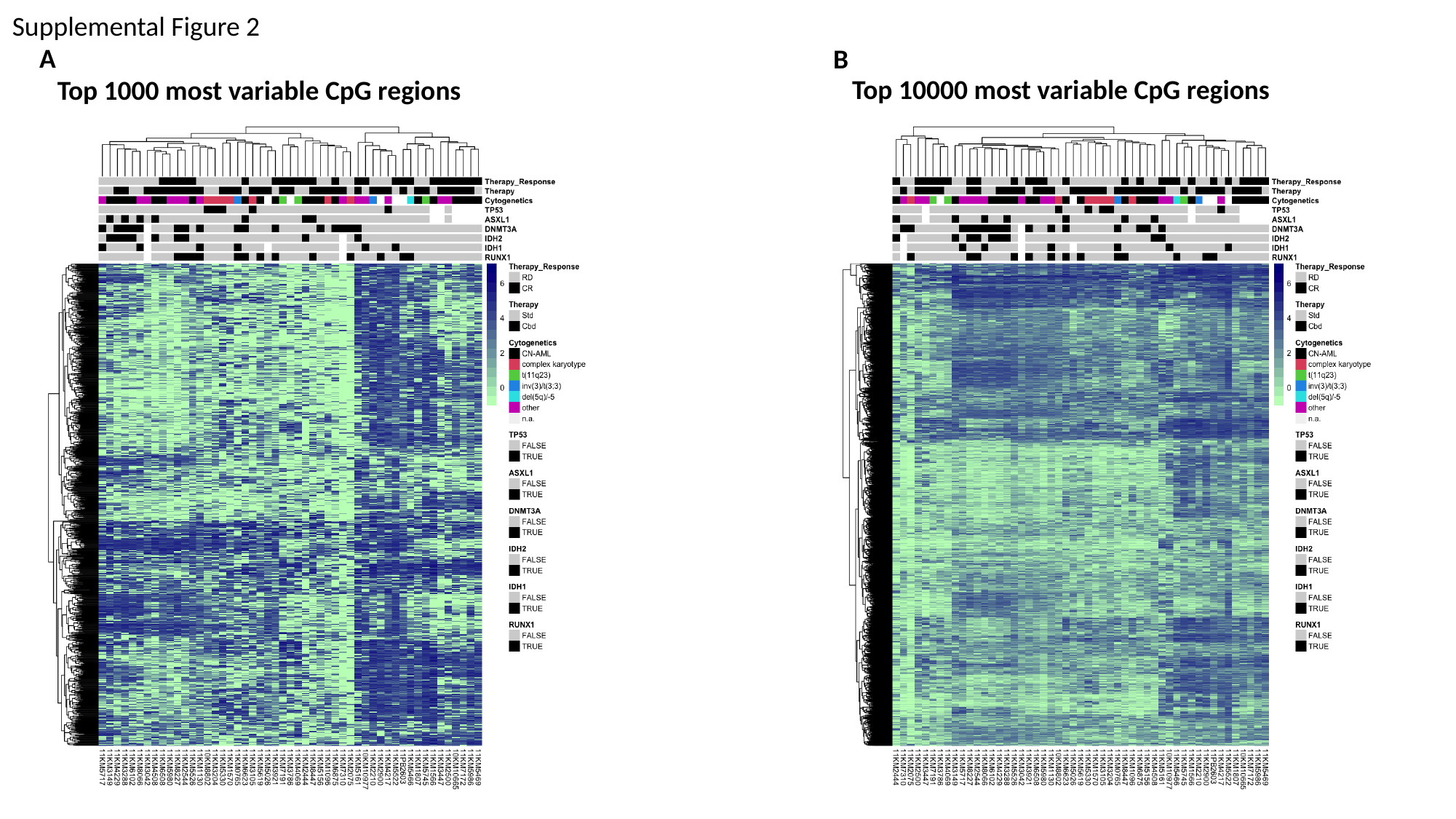

Supplemental Figure 2
A
B
Top 10000 most variable CpG regions
Top 1000 most variable CpG regions

## Slide 3
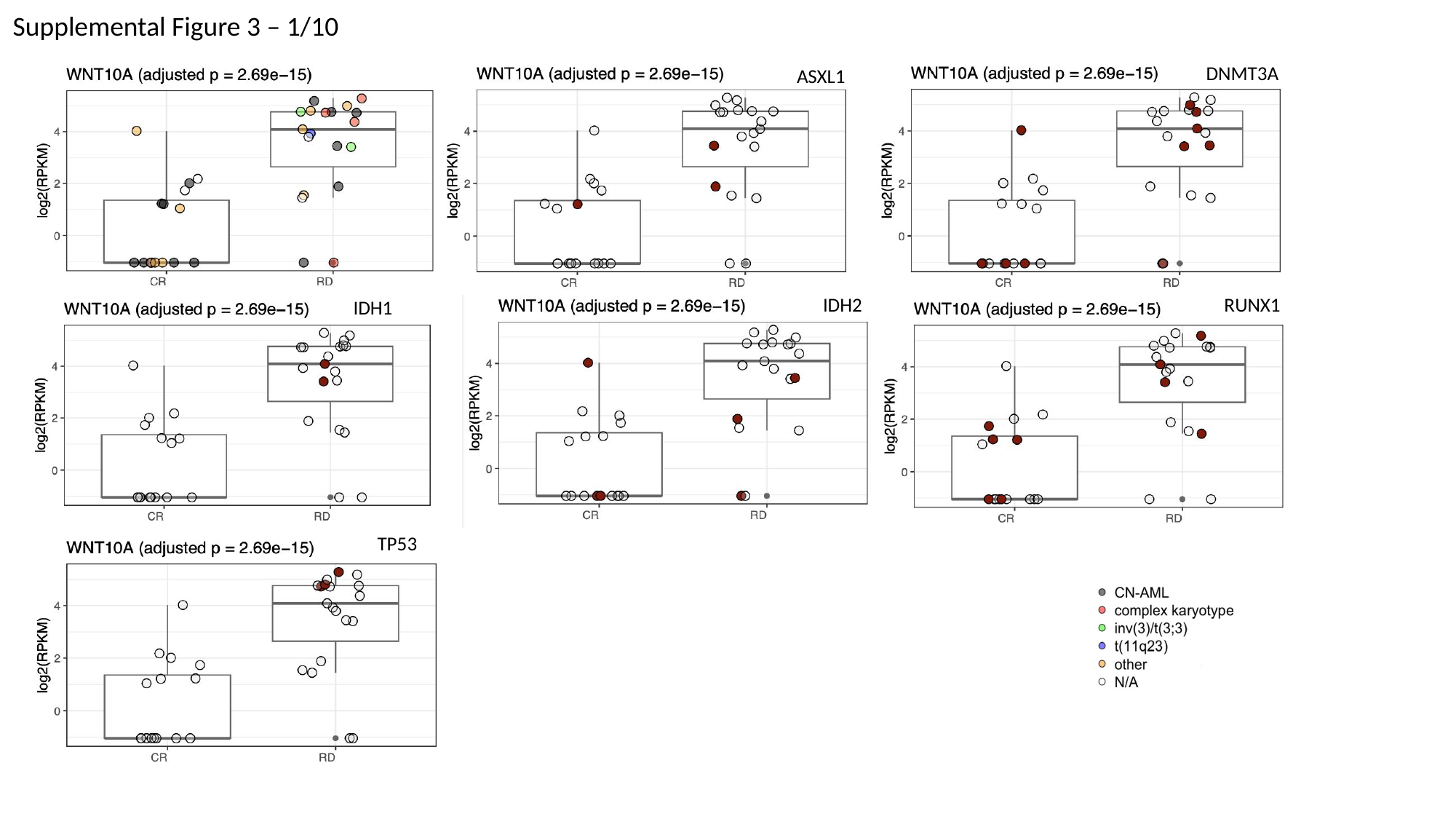

Supplemental Figure 3 – 1/10
DNMT3A
ASXL1
IDH2
RUNX1
IDH1
TP53

## Slide 4
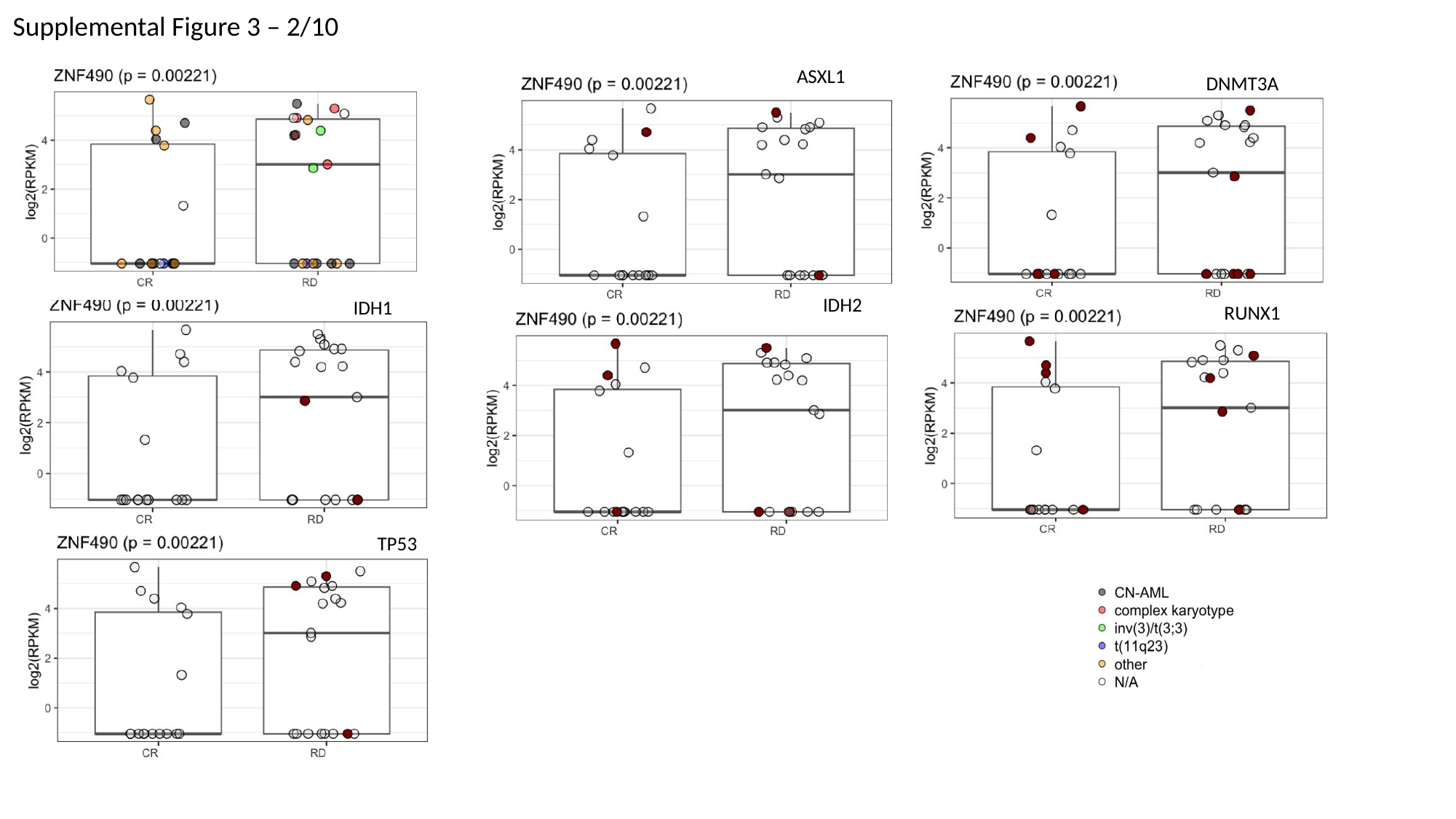

Supplemental Figure 3 – 2/10
ASXL1
DNMT3A
IDH2
IDH1
RUNX1
TP53

## Slide 5
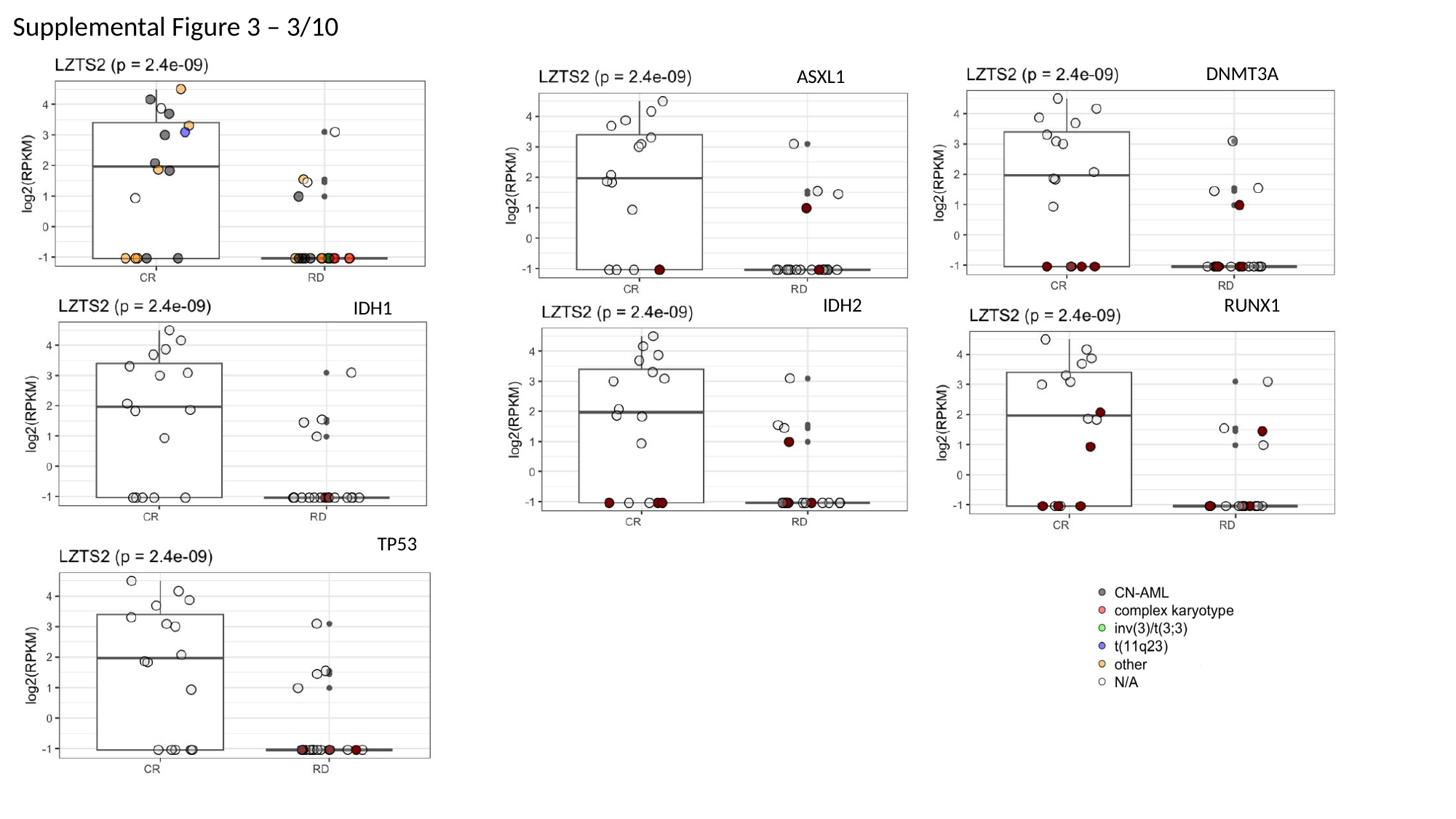

Supplemental Figure 3 – 3/10
DNMT3A
ASXL1
IDH2
RUNX1
IDH1
TP53

## Slide 6
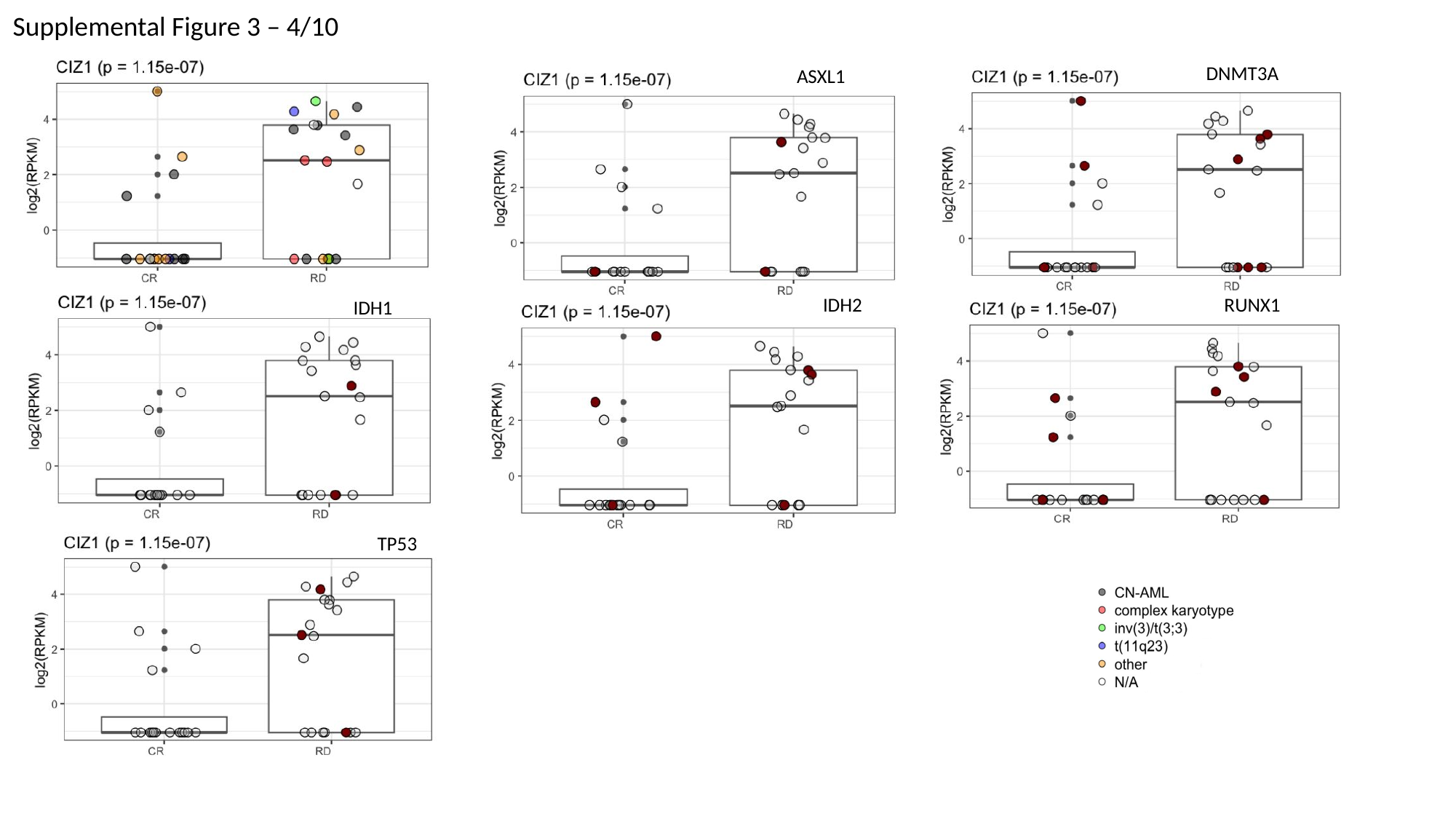

Supplemental Figure 3 – 4/10
DNMT3A
ASXL1
IDH2
RUNX1
IDH1
TP53

## Slide 7
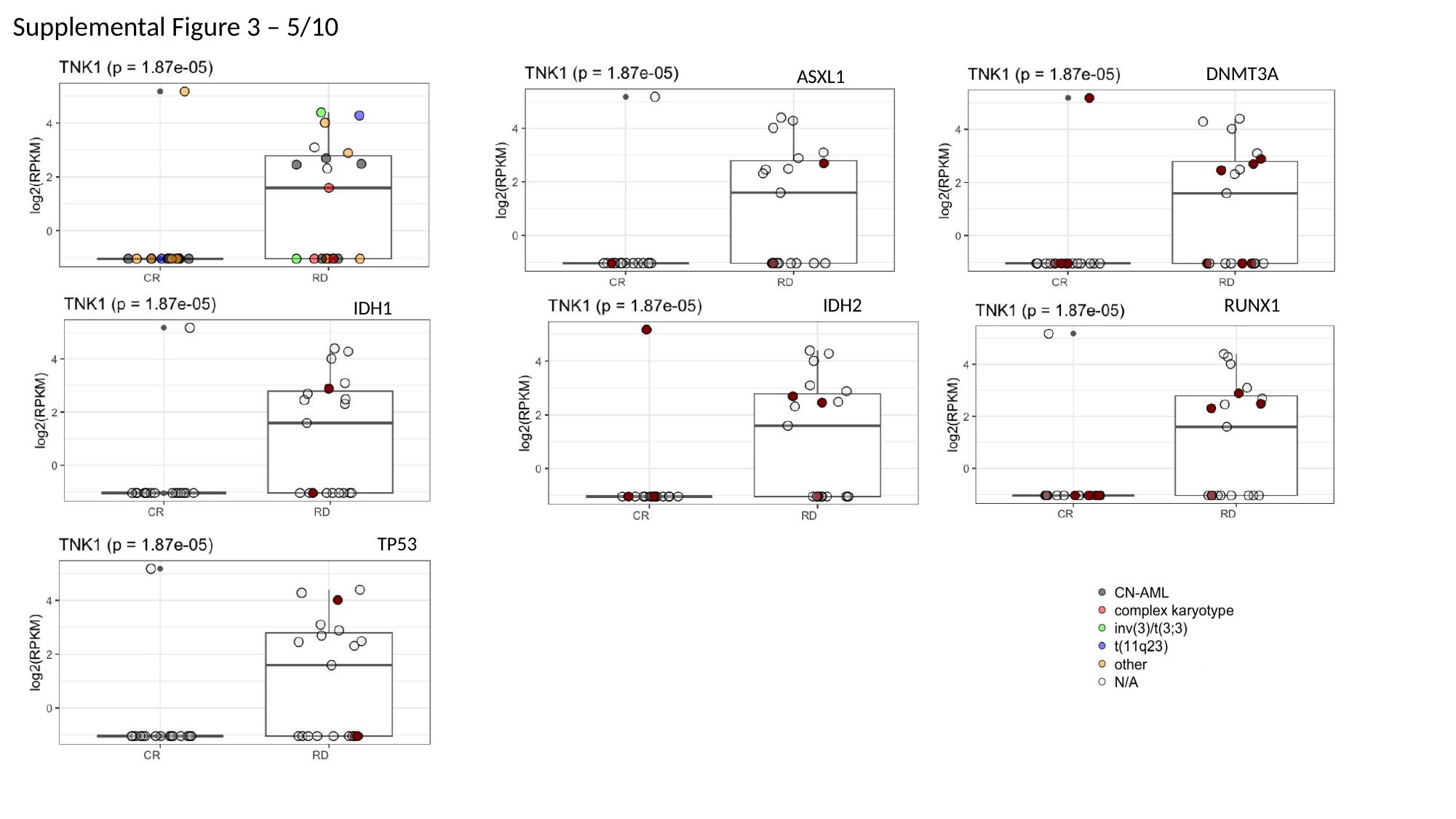

Supplemental Figure 3 – 5/10
DNMT3A
ASXL1
IDH2
RUNX1
IDH1
TP53

## Slide 8
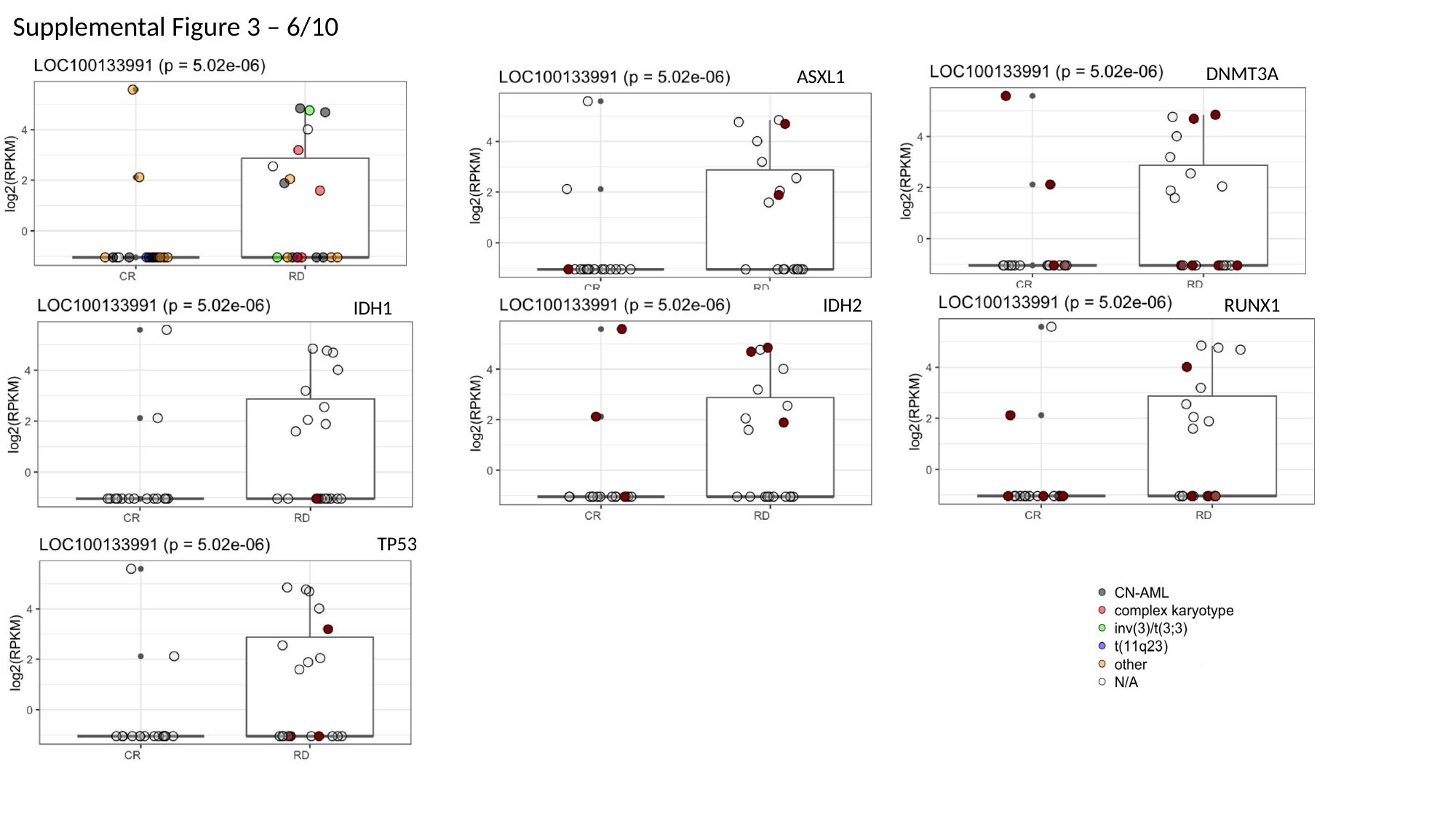

Supplemental Figure 3 – 6/10
DNMT3A
ASXL1
IDH2
RUNX1
IDH1
TP53

## Slide 9
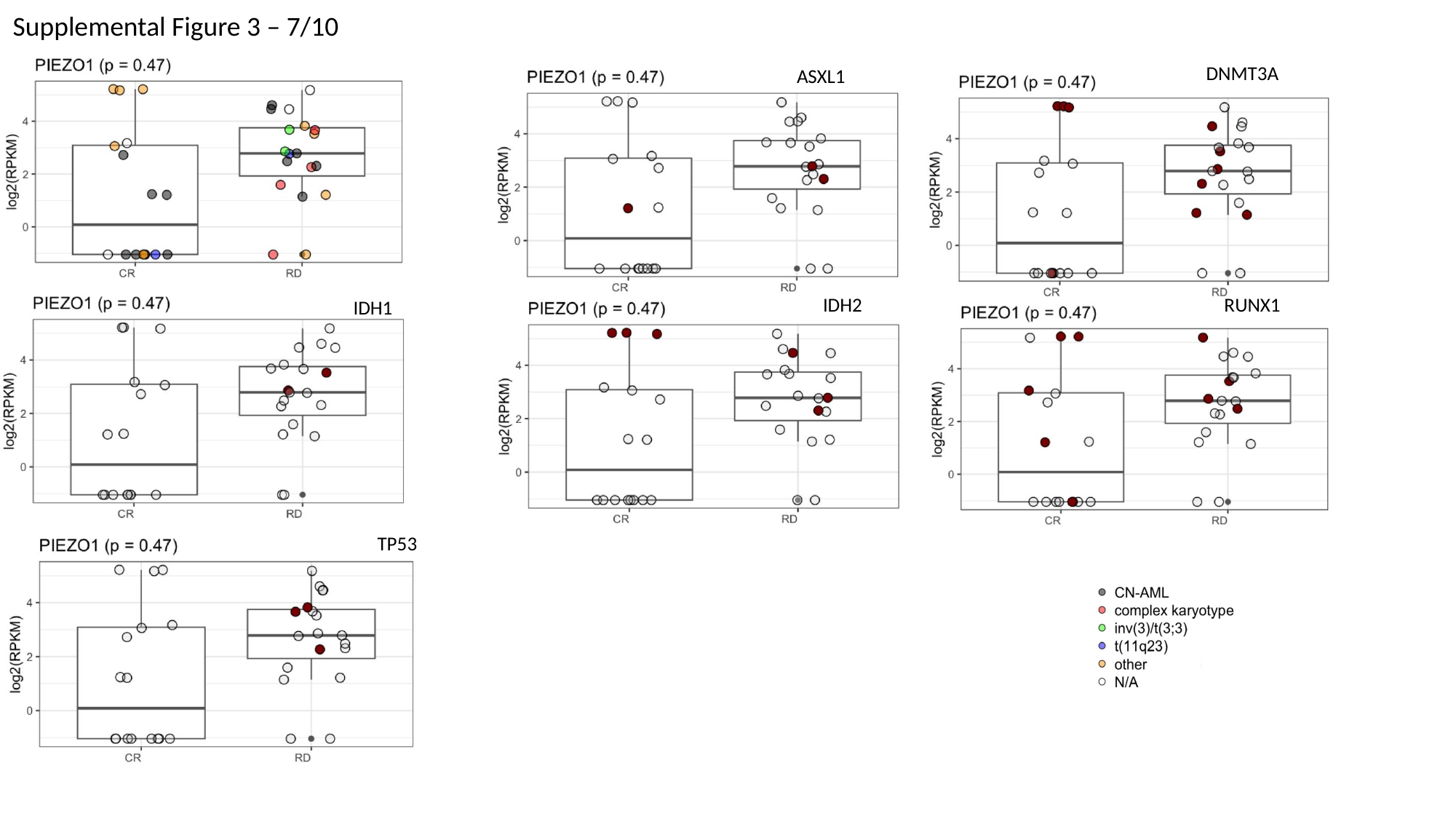

Supplemental Figure 3 – 7/10
DNMT3A
ASXL1
IDH2
RUNX1
IDH1
TP53

## Slide 10
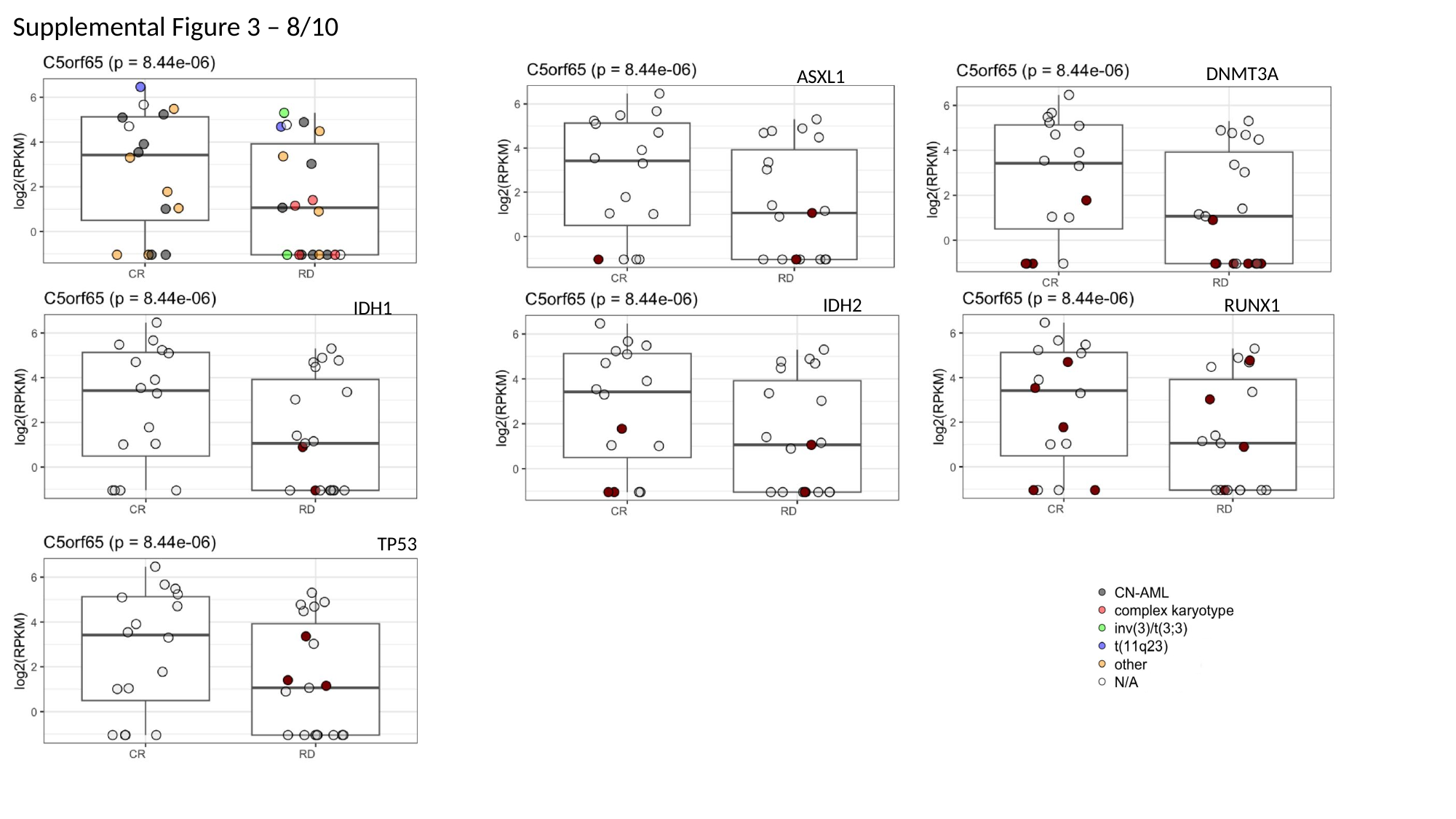

Supplemental Figure 3 – 8/10
DNMT3A
ASXL1
IDH2
RUNX1
IDH1
TP53

## Slide 11
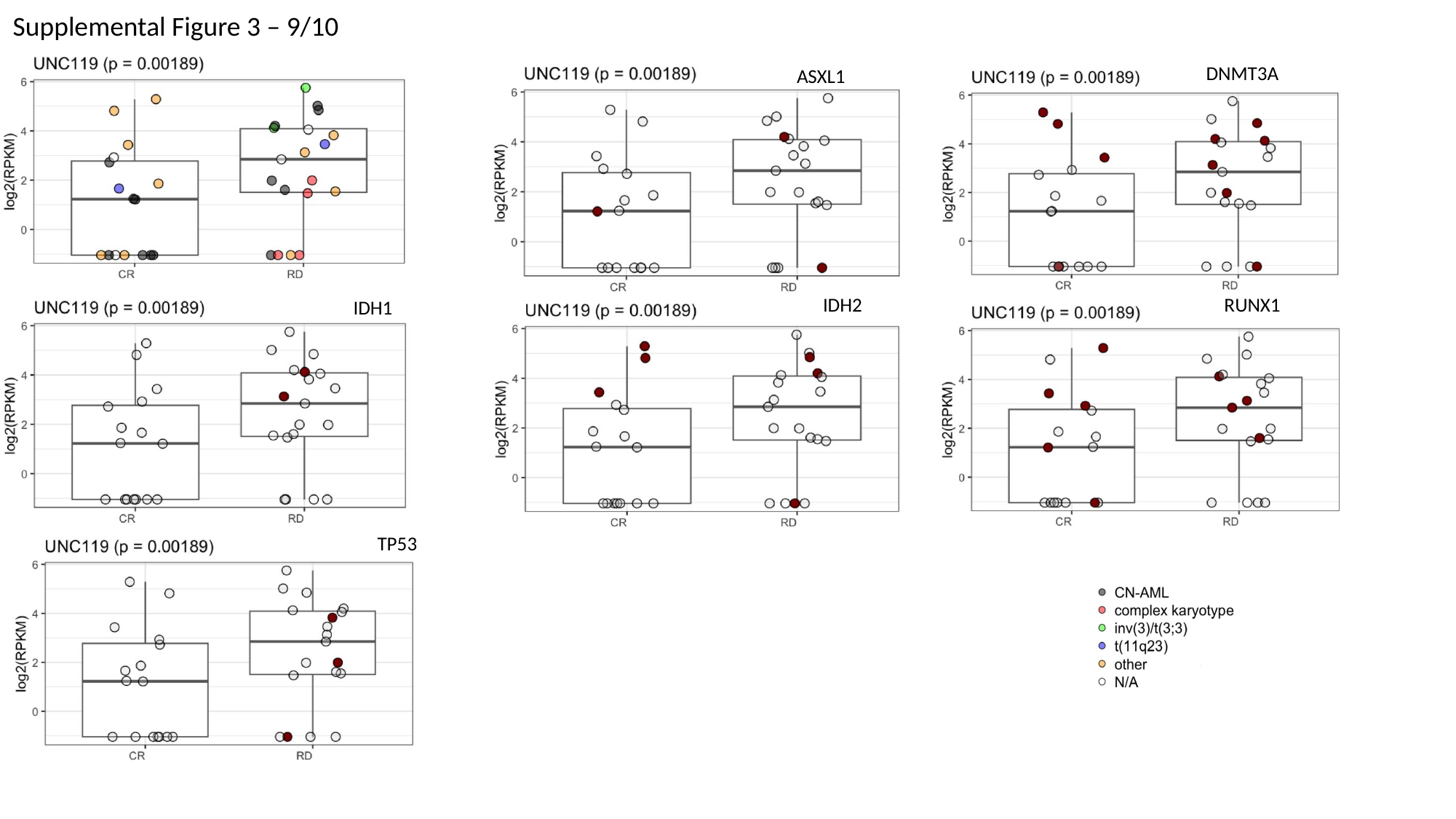

Supplemental Figure 3 – 9/10
DNMT3A
ASXL1
IDH2
RUNX1
IDH1
TP53

## Slide 12
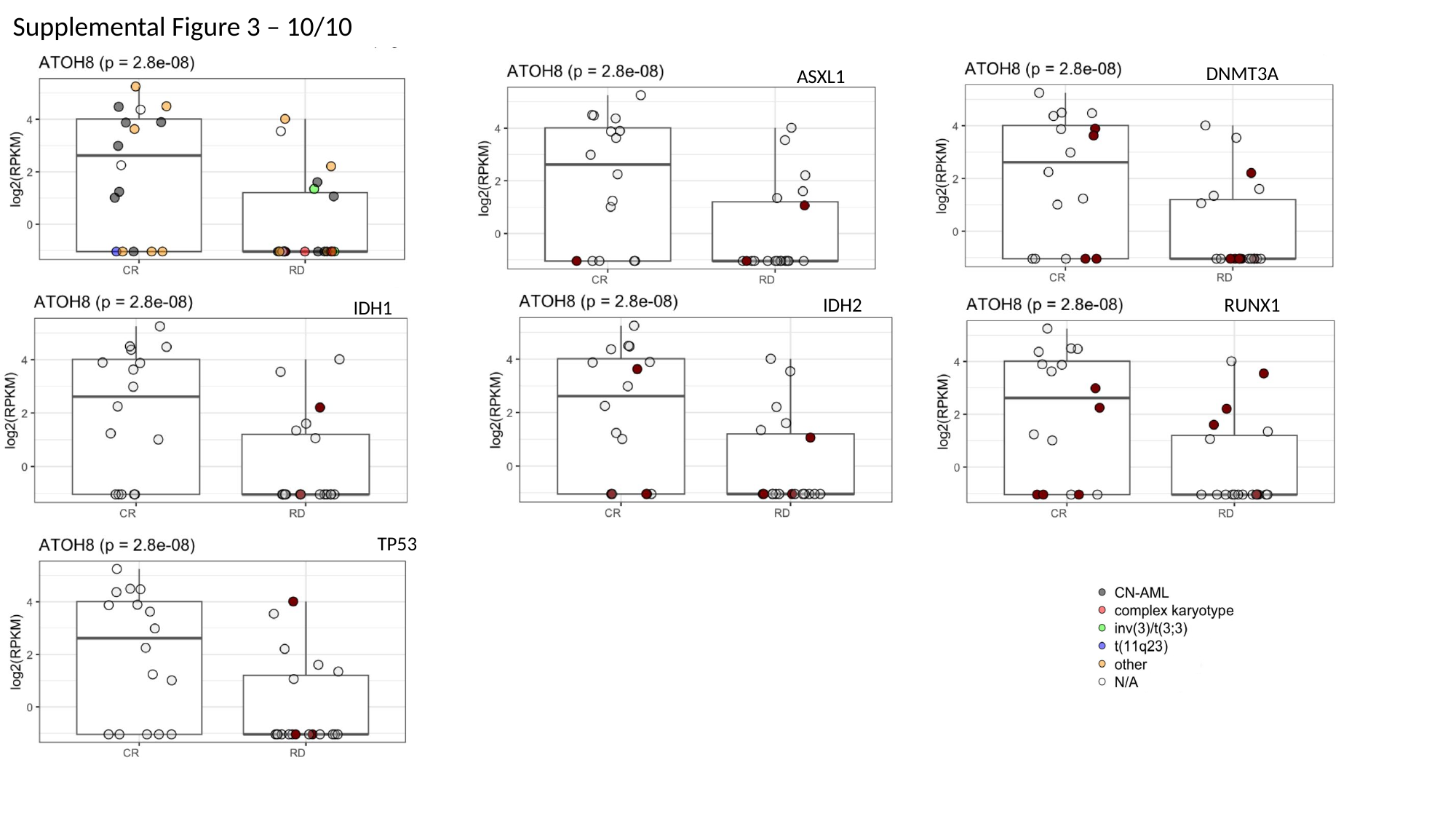

Supplemental Figure 3 – 10/10
DNMT3A
ASXL1
IDH2
RUNX1
IDH1
TP53

## Slide 13
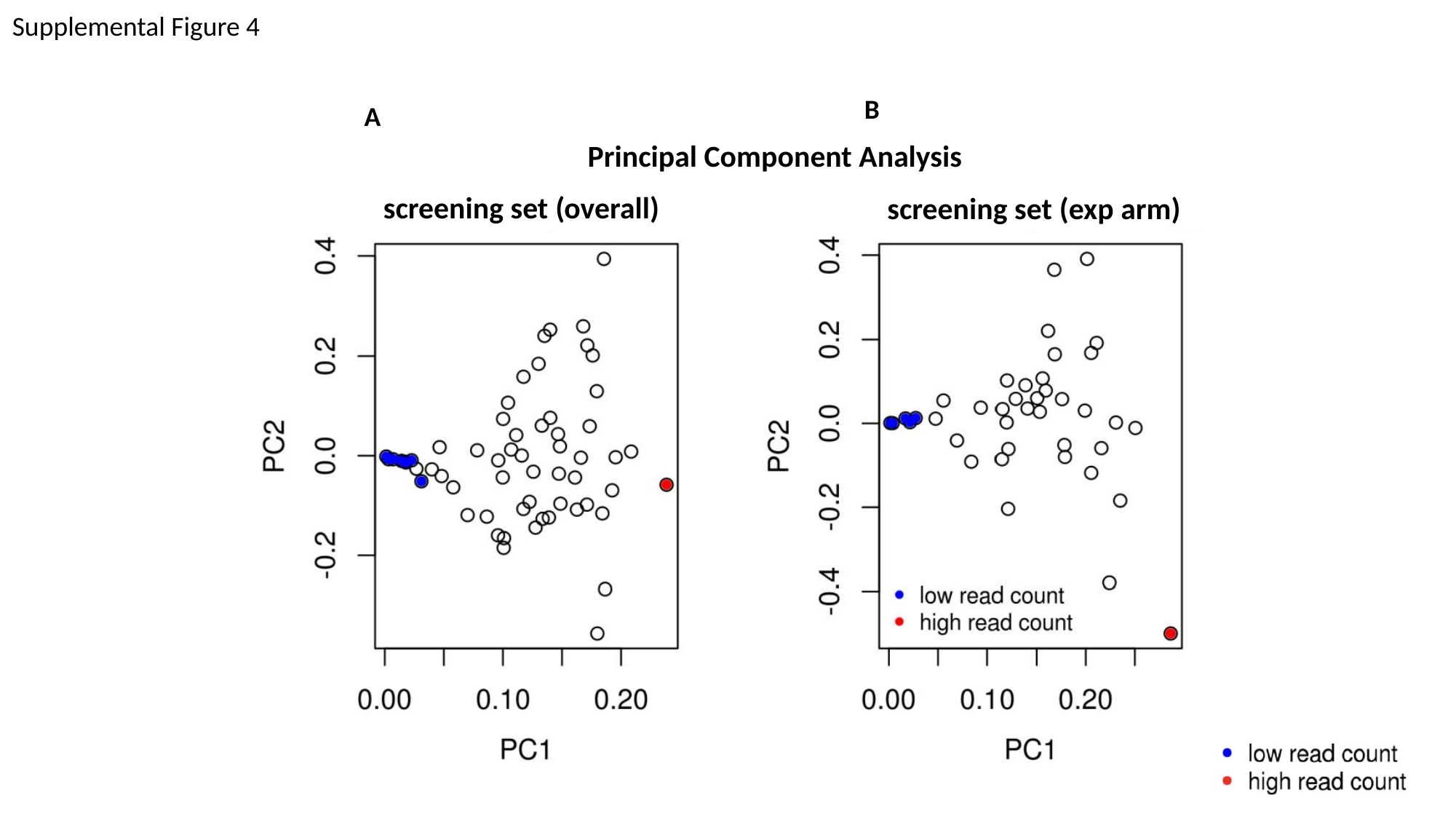

Supplemental Figure 4
B
A
Principal Component Analysis
screening set (overall)
screening set (exp arm)

## Slide 14
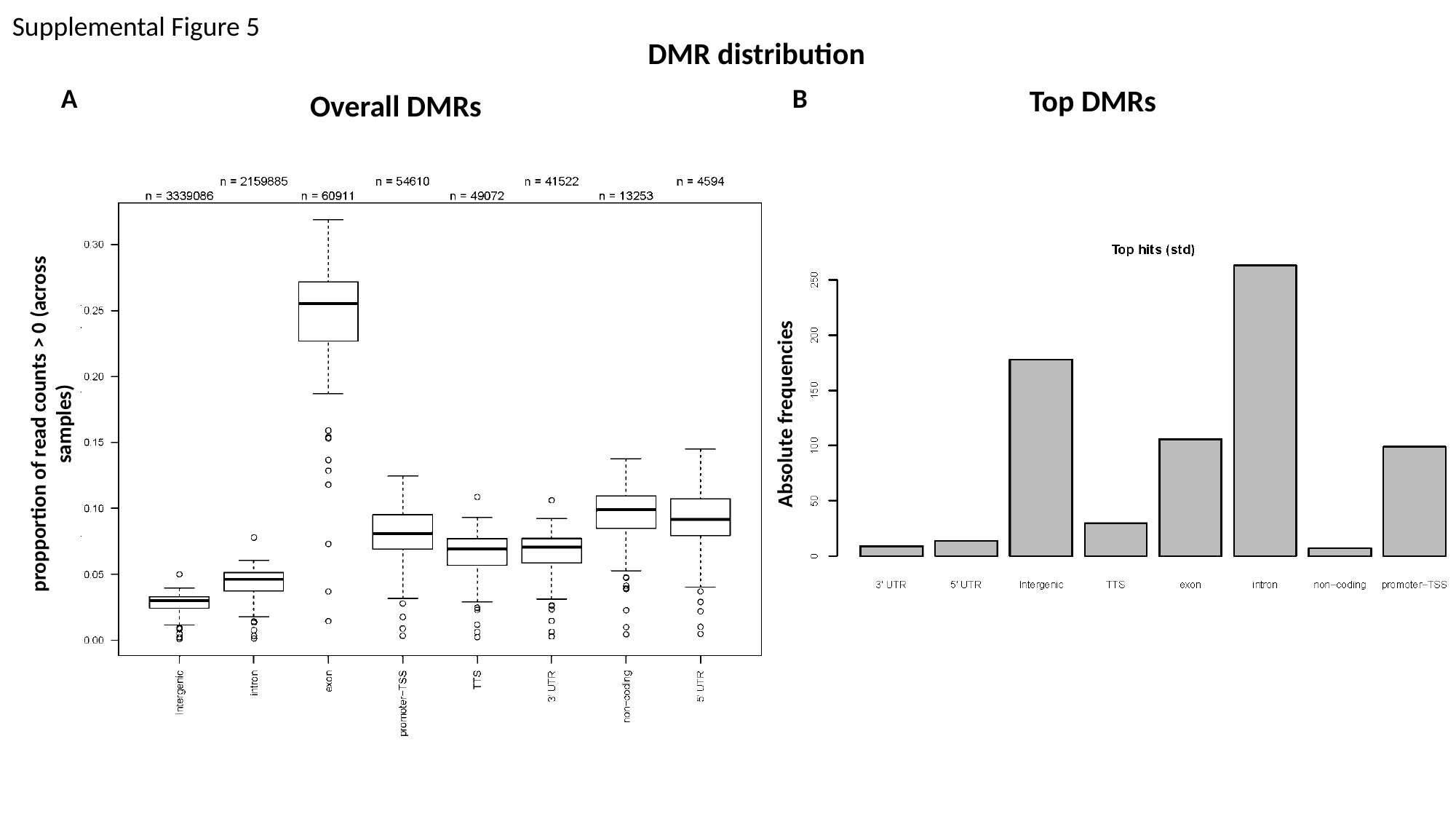

Supplemental Figure 5
DMR distribution
A
B
Top DMRs
Overall DMRs
propportion of read counts > 0 (across samples)
Absolute frequencies

## Slide 15
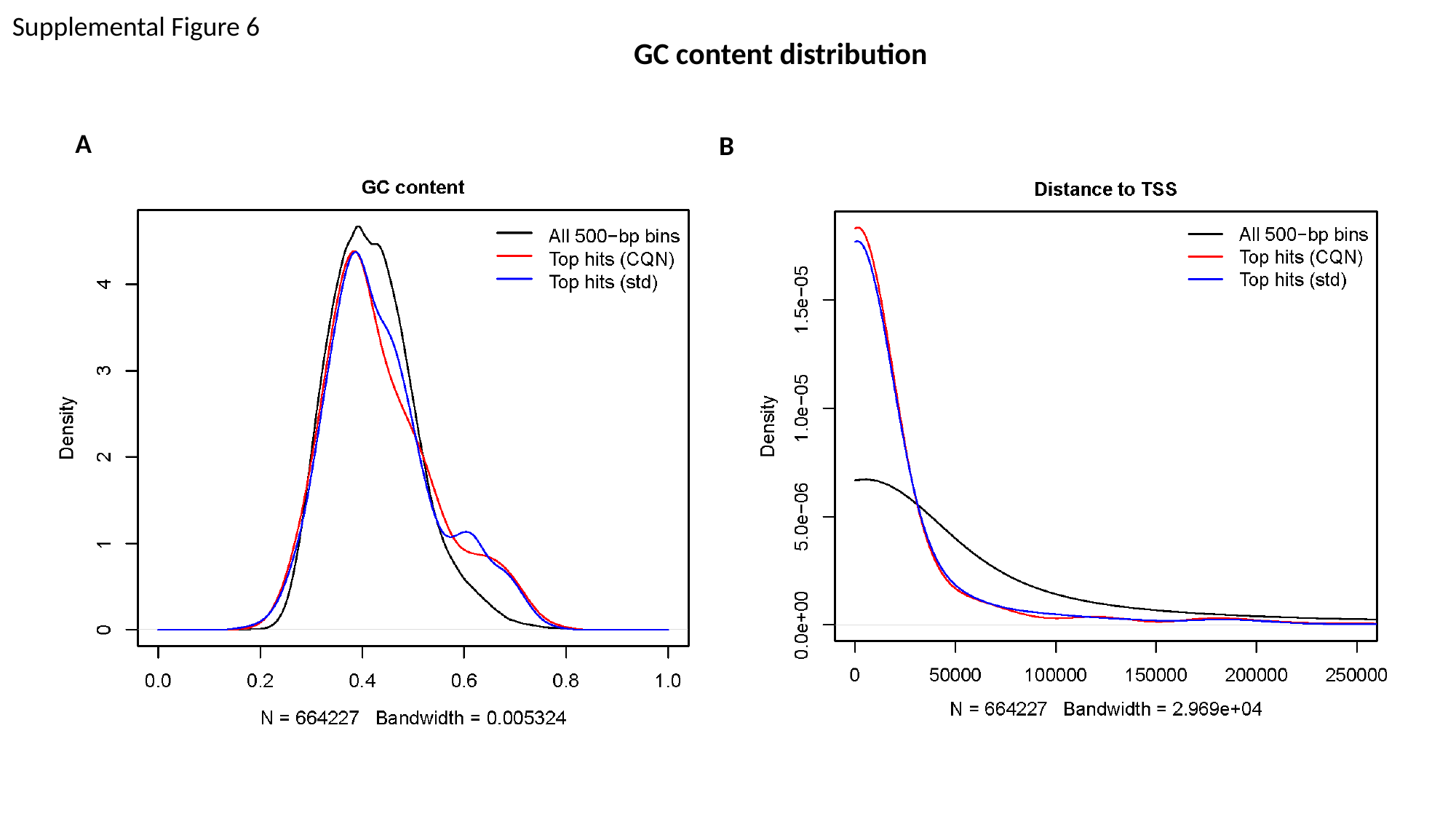

Supplemental Figure 6
GC content distribution
A
B

## Slide 16
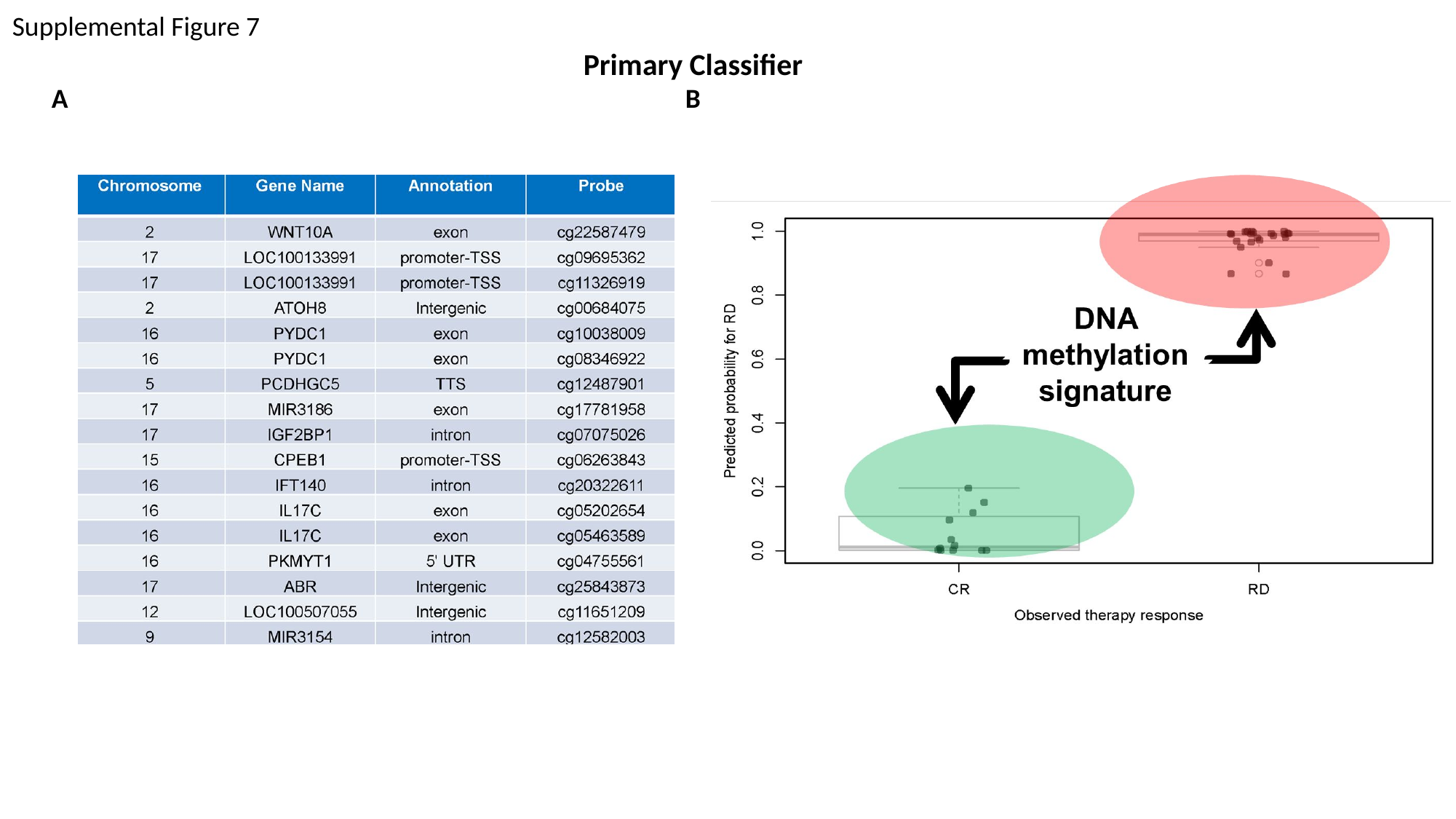

Supplemental Figure 7
Primary Classifier
A
B

## Slide 17
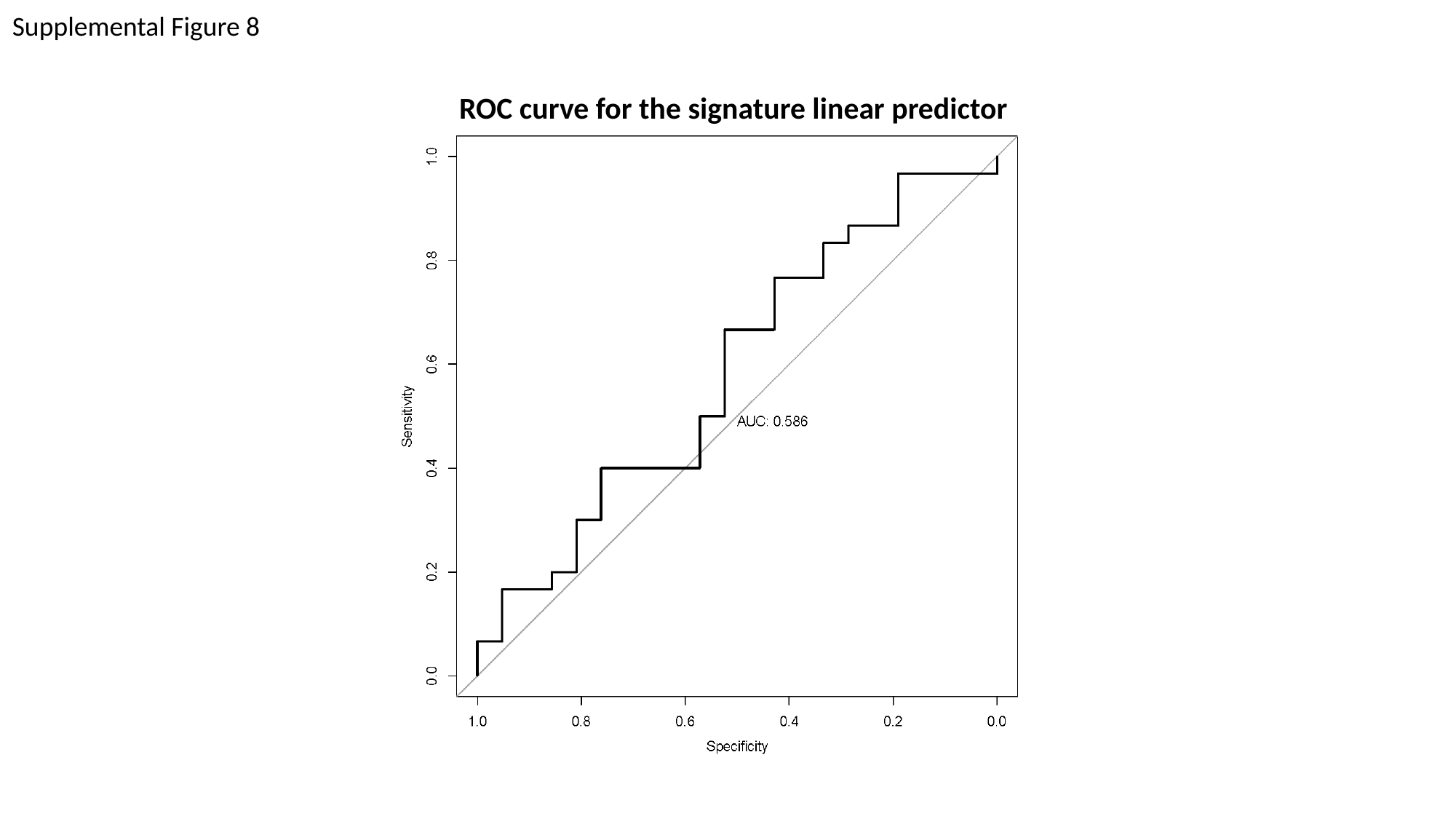

Supplemental Figure 8
ROC curve for the signature linear predictor

## Slide 18
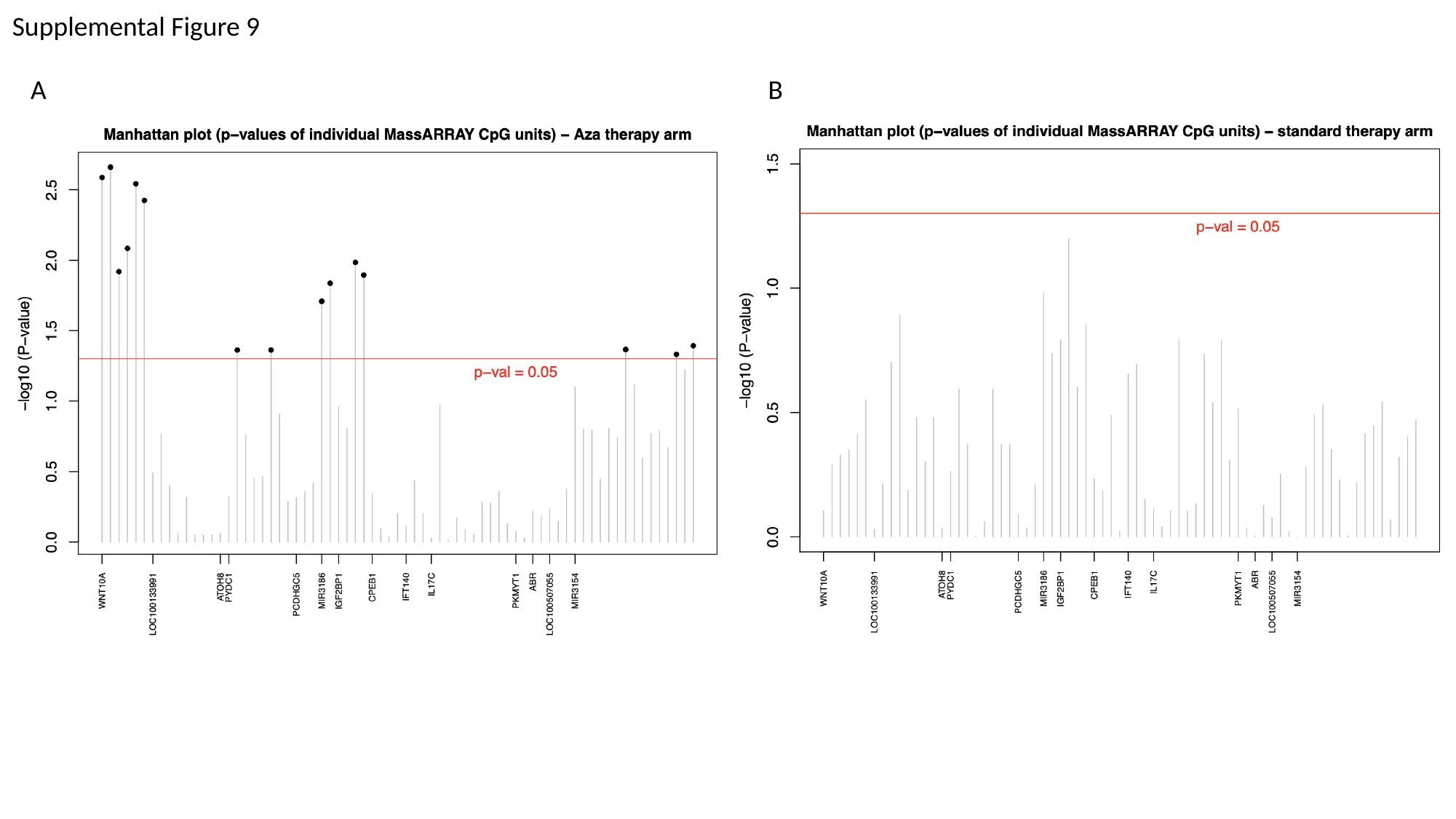

Supplemental Figure 9
A
B

## Slide 19
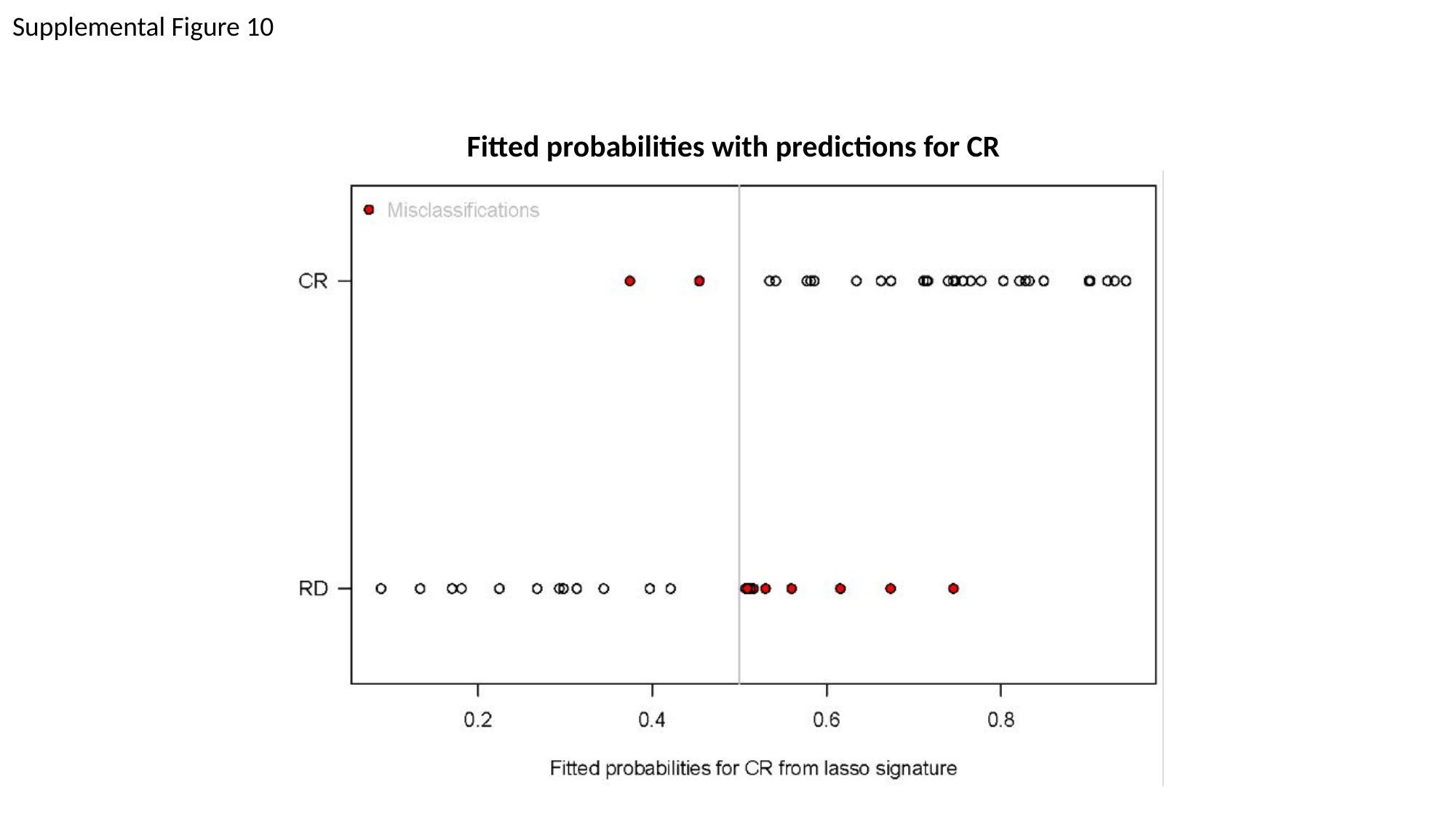

Supplemental Figure 10
Fitted probabilities with predictions for CR

## Slide 20
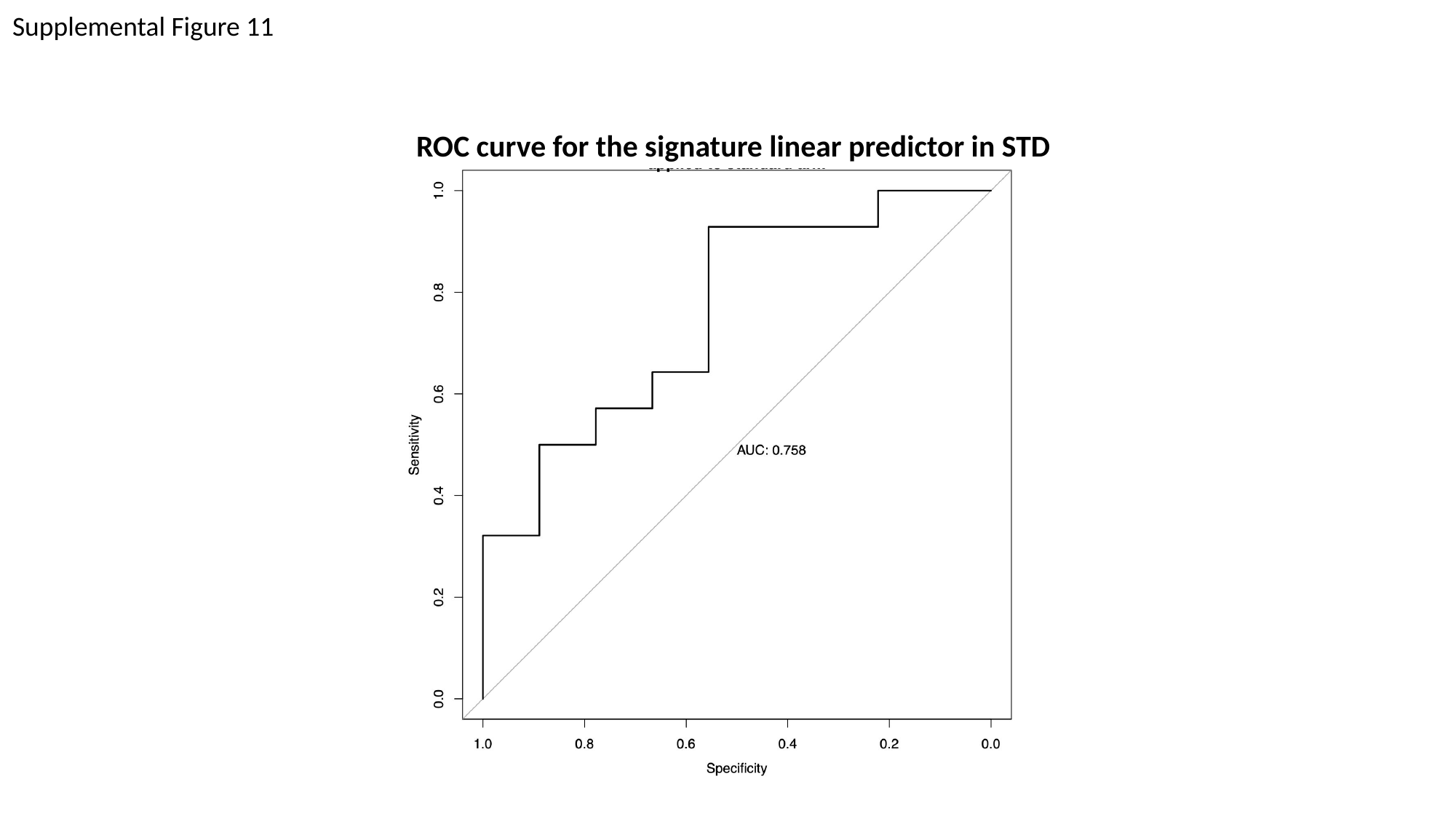

Supplemental Figure 11
ROC curve for the signature linear predictor in STD
