# Supplementary material for: Predictive value of DNA methylation patterns in AML patients treated with an azacytidine containing induction regimen
Source: Clin Epigenetics. 2023 Oct 26;15:171. doi: 10.1186/s13148-023-01580-z (PMC10601277; doi:10.1186/s13148-023-01580-z)
Supplement: Supplementary file 3 — Additional file 3. Overview of predictive epigenetic biomarkers. The table provides a short review of published predictive biomarkers related to DNA-methylation and hypomethylating agents. [file 13148_2023_1580_MOESM3_ESM.docx]

| **Title, Journal, Author** | **Year** | **DOI** | **Methods** | **Key Findings** |
| --- | --- | --- | --- | --- |
| [1] Welch, J.S., et al., *TP53 and Decitabine in Acute Myeloid Leukemia and Myelodysplastic Syndromes.* N Engl J Med, 2016. **375**(21): p. 2023-2036. | 2016 | 10.1056/NEJMoa1605949 | - Single institutional study with n = 84 AML and MDS patients with a 10-day DAC 20 mg/m^2^ regimen - included enhanced exome / gene panel sequencing | - DAC response rates were higher among patients with an unfavorable-risk cytogenetic profile (67% vs 34%) - and higher among patients with *tp53*-mutations (100% vs 41%) - but the responses were not durable and OS rates were similar to patients with an intermediate-risk cytogenetic profile - unclear if 5-day or 10-day regimen is to be recommended |
| [2] Meldi, K., et al., *Specific molecular signatures predict decitabine response in chronic myelomonocytic leukemia.* J Clin Invest, 2015. **125**(5): p. 1857-72. | 2015 | 10.1172/JCI78752 | - EERBS - 40 CMML patients treated with Decitabine - somatic mutations did not differentiate R from NR - identification of 167 DMRs with NGS - transcriptional analysis revealed diff. in gene expression transcriptional analysis revealed diff. in gene expression | - DMRs localized in nonpromoter regions and overlapped with distal reg. enhacers - using methalytion profiles, an epigenetic classifier was developed; upregulated genes were ass. with cell cycle (pot. mech in CAR incorporation) |
| [3] Shen, L., et al., *DNA methylation predicts survival and response to therapy in patients with myelodysplastic syndromes.* J Clin Oncol, 2010. **28**(4): p. 605-13. | 2010 | 10.1200/JCO.2009.23.4781 | - “Screened” for promoter CpG island methylation of 24 genes in 24 patients - Screening = individual selection of known genes + genes via own approach (genomewide meth CpG island ampl./repr. difference analysis) - Found aberrant hypermethylation in 10 genes - Quantitative analysis of identified genes in 317 patients (**bisulfite pyrosequencing**) | - Higher methylation as prognostic marker (outcome inferior) - Validated in 2 independent cohorts - methylation at baseline did not correlate with clinical response to HMA - but found correlation between reduced methylation over time and clinical response - CpG islands have been suggested being a biomarker predicting response to HMAs |
| [4] Fandy, T.E., et al., *Early epigenetic changes and DNA damage do not predict clinical response in an overlapping schedule of 5-azacytidine and entinostat in patients with myeloid malignancies.* Blood, 2009. **114**(13): p. 2764-73. | 2009 | 10.1182/blood-2009-02-203547. | - methylation-specific PCR | - presumed tumor suppressor genes (CDKN2B, CDH1, DAPK1, and SOCS1) in patients treated with AZA and etinostat, a histone deacetylase inhibitor, no correlation was detected between clinical response, baseline methylation, changes in methylation or gene expression |
| [5] Hiller, J.K., et al., *Evaluating the impact of genetic and epigenetic aberrations on survival and response in acute myeloid leukemia patients receiving epigenetic therapy.* Ann Hematol, 2017. **96**(4): p. 559-565. | 2017 | <https://doi.org/10.1007/s00277-016-2912-7> | - targeted bisulfite pyrosequencing - in 87 AML patients from the AML00331 trial, distinct genetic (FLT3, NPM, DNMT3A) and epigenetic (ERa, CEBPA OLIG2) aberrations were analysed) | - Another study analyzing DNA promoter methylation of DAC treated AML patients revealed an association towards decreased survival of patients with increased methylation levels of ESR1 (p=0.07) and OLIG2 (p=0.08). In multivariable analysis adjusted for clinical baseline parameters, the impact of ESR1 and OLIG2 methylation was retained - none of the investigated genetic and epigenetic markers was associated with response to treatment |
| [6] Yan, P., et al., *Genome-wide methylation profiling in decitabine-treated patients with acute myeloid leukemia.* Blood, 2012. **120**(12): p. 2466-74. | 2012 | 10.1182/blood-2012-05-429175 | - MethylCapSeq | - genome wide methylation study using MethylCap-seq in AML patients receiving DAC, has shown that a higher baseline methylation level and more distinctive methylation decrease were associated with response |
| [7] Papaemmanuil, E., et al., *Clinical and biological implications of driver mutations in myelodysplastic syndromes.* Blood, 2013. **122**(22): p. 3616-27; quiz 3699. | 2013 | 10.1182/blood-2013-08-518886 | - 111 select gene panel via Illumina HiSeq across 738 patients with MDS/CMML | - Neither the measurement of pre-treatment DNA methylation nor the presence of TP53 mutations is predictive for response to azacitidine in MDS |
| [8] Traina, F., et al., *Impact of molecular mutations on treatment response to DNMT inhibitors in myelodysplasia and related neoplasms.* Leukemia, 2014. **28**(1): p. 78-87. | 2014 | 10.1038/leu.2013.269 | - n=230 patients included - hypothesis: mutations in MDS are predictive biomarkers - **Sanger-Seq** for spec. mutations | - In multivariate analysis, TET2MUT and/or DNMT3AMUT, platelets >= 100 and WBC <3 were independent predictors of better response. - Strikingly (not mentioned): no mutational pattern identified, that is associated with non-repsonse: non-responders cannot be identified via specific mutations |
| [9] Emadi, A., et al., Presence of isocitrate dehydrogenase mutations may predict clinical response to hypomethylating agents in patients with acute myeloid leukemia. Am J Hematol, 2015. 90(5): p. E77-9. | 2015 | 10.1002/ajh.23965 | - Retrospective analysis of AML patients with IDH mutations (Sanger Seq) | - Odds 14,2 for response after HMA administration in IDH mutated patients |
| [10] Zhang, L.Y., et al., *Impact of Global and Gene-Specific DNA Methylation in de Novo or Relapsed Acute Myeloid Leukemia Patients Treated with Decitabine.* Asian Pac J Cancer Prev, 2016. **17**(1): p. 431-7. | 2016 | 10.7314/apjcp.2016.17.1.431 | - global DNA methylation assessment via ELISA - methylation assessment of 5 genes via PCR - PB-DNA of 42 patients analysed on days -1,1,7 | - higher methylation ass. with poorer prognosis after treatment - higher methylation of sox2 oct4 associated with differential response to decitabine |

1. Welch, J.S., et al., *TP53 and Decitabine in Acute Myeloid Leukemia and Myelodysplastic Syndromes.* N Engl J Med, 2016. **375**(21): p. 2023-2036.

2. Meldi, K., et al., *Specific molecular signatures predict decitabine response in chronic myelomonocytic leukemia.* J Clin Invest, 2015. **125**(5): p. 1857-72.

3. Shen, L., et al., *DNA methylation predicts survival and response to therapy in patients with myelodysplastic syndromes.* J Clin Oncol, 2010. **28**(4): p. 605-13.

4. Fandy, T.E., et al., *Early epigenetic changes and DNA damage do not predict clinical response in an overlapping schedule of 5-azacytidine and entinostat in patients with myeloid malignancies.* Blood, 2009. **114**(13): p. 2764-73.

5. Hiller, J.K., et al., *Evaluating the impact of genetic and epigenetic aberrations on survival and response in acute myeloid leukemia patients receiving epigenetic therapy.* Ann Hematol, 2017. **96**(4): p. 559-565.

6. Yan, P., et al., *Genome-wide methylation profiling in decitabine-treated patients with acute myeloid leukemia.* Blood, 2012. **120**(12): p. 2466-74.

7. Papaemmanuil, E., et al., *Clinical and biological implications of driver mutations in myelodysplastic syndromes.* Blood, 2013. **122**(22): p. 3616-27; quiz 3699.

8. Traina, F., et al., *Impact of molecular mutations on treatment response to DNMT inhibitors in myelodysplasia and related neoplasms.* Leukemia, 2014. **28**(1): p. 78-87.

9. Emadi, A., et al., *Presence of isocitrate dehydrogenase mutations may predict clinical response to hypomethylating agents in patients with acute myeloid leukemia.* Am J Hematol, 2015. **90**(5): p. E77-9.

10. Zhang, L.Y., et al., *Impact of Global and Gene-Specific DNA Methylation in de Novo or Relapsed Acute Myeloid Leukemia Patients Treated with Decitabine.* Asian Pac J Cancer Prev, 2016. **17**(1): p. 431-7.
