# Supplementary material for: Predictive value of DNA methylation patterns in AML patients treated with an azacytidine containing induction regimen
Source: Clin Epigenetics. 2023 Oct 26;15:171. doi: 10.1186/s13148-023-01580-z (PMC10601277; doi:10.1186/s13148-023-01580-z)
Supplement: Supplementary file 4 — Additional file 4. Patient characteristics within the screening cohort both for the standard arm as well as for the experimental arm are provided. [file 13148_2023_1580_MOESM4_ESM.docx]

**Supplemental Table 2 – Differences in patient characteristics within screening cohort**

| **Characteristic** | **STD** | **EXP** | **p-value** |
| --- | --- | --- | --- |
| **Age – year *** | **59.6 ± 3.4** | **61.0 ± 1.8** | **0.70** |
| **Female sex – no. (%)** | **9 (50)** | **21 (53)** | **> 0.99** |
| **Complete response – no. (%)** | **10 (56)** | **19 (48)** | **0.78** |
| **Cytogenetic grouping – no (%)**  **normal karyotype**  **complex karyotype**  **del(5q)/-5**  **inv(3)/t(3;3)**  **t(11q23)**  **other**  **missing** | **7 (39)**  **3 (17)**  **1 (6)**  **0 (0)**  **1 (5,6)**  **4 (22)**  **2 (11)** | **14 (35)**  **6 (15)**  **0 (0)**  **2 (5)**  **2 (5)**  **11 (28)**  **5 (13)** | **0.77**  **0.87**  **0.14**  **0.97**  **0.93**  **0.67**  **0.88** |
| **White blood cell counts (G/l) **** | **3.7** | **5.3** | **0.16** |
| **Peripheral blood blasts (%) **** | **17.5** | **18** | **0.96** |
| **Bone marrow blasts (%) **** | **50.0** | **66.5** | **0.02** |

*Plus-minus values are means ± SD. P Values are for the overall comparisons

** Median values are given
