# Supplementary material for: Predictive value of DNA methylation patterns in AML patients treated with an azacytidine containing induction regimen
Source: Clin Epigenetics. 2023 Oct 26;15:171. doi: 10.1186/s13148-023-01580-z (PMC10601277; doi:10.1186/s13148-023-01580-z)
Supplement: Supplementary file 5 — Additional file 5. Overview of top differentially methylated regions within the experimental treatment arm. Selected candidate regions are marked in green. Logarithmic fold change (logFC) and p-values are given for all regions. Respective values form the standard arm are highlighted in yellow. All regions are arranged in ascending order of p-values (experimental arm). [file 13148_2023_1580_MOESM5_ESM.docx]

# Chr Start End Annotation Detailed.Annotation Gene.Name gc.conte logFC PValue FDR

1 chr3 128205501 128206001 exon exon (NM_001145662, exon 2 of 6) GATA2 0,283 -5,475 3,38E-20 2,26E-14

2 chr2 219738001 219738501 exon exon (NM_006522, exon 4 of 4) WNT10A 0,248 3,565 2,14E-17 7,14E-12

3 chr9 131413501 131414001 intron AluSz|SINE|Alu WDR34 0,443 -7,655 4,41E-16 9,84E-11

4 chr19 12698501 12699001 intron AluSq2|SINE|Alu ZNF490 0,411 2,652 5,65E-14 9,44E-09

5 chr10 8097501 8098001 exon exon (NM_002051, exon 2 of 6) GATA3 0,403 7,800 1,52E-13 2,03E-08

6 chr22 19710501 19711001 promoter-TSS promoter-TSS (NM_000407) GP1BB 0,401 2,793 2,11E-13 2,36E-08

7 chr10 102760001 102760501 intron intron (NM_032429, intron 1 of 4) LZTS2 0,543 -3,583 2,53E-13 2,42E-08

8 chr20 44098501 44099001 intron CpG WFDC2 0,451 5,245 5,70E-13 4,76E-08

9 chr19 10406001 10406501 exon exon (NM_003259, exon 10 of 11) ICAM5 0,511 7,675 9,71E-13 7,21E-08

10 chr19 39360001 39360501 exon exon (NM_001195833, exon 10 of 12) RINL 0,359 7,613 3,07E-12 2,06E-07

11 chr7 27205001 27205501 promoter-TSS promoter-TSS (NM_152739) HOXA9 0,353 -5,334 4,94E-12 3,01E-07

12 chr3 129147001 129147501 intron CpG C3orf25 0,405 2,787 5,46E-12 3,05E-07

13 chr9 130981001 130981501 intron CpG CIZ1 0,525 3,964 6,04E-12 3,11E-07

14 chr17 7287001 7287501 intron CpG TNK1 0,405 7,554 7,37E-12 3,52E-07

15 chr17 43324501 43325001 promoter-TSS promoter-TSS (NR_024434) LOC100133991 0,343 4,352 8,89E-12 3,87E-07

16 chr7 30028001 30028501 intron (CA)n|Simple_repeat|Simple_repeat SCRN1 0,365 -1,740 9,67E-12 3,87E-07

17 chr1 36786501 36787001 TTS TTS (NM_018166) FAM176B 0,685 3,930 9,98E-12 3,87E-07

18 chr16 87309501 87310001 Intergenic Intergenic C16orf95 0,395 -2,816 1,04E-11 3,87E-07

19 chr3 194868501 194869001 intron intron (NM_152531, intron 3 of 3) XXYLT1 0,453 -2,288 1,15E-11 4,05E-07

20 chr22 46440001 46440501 intron intron (NR_027036, intron 1 of 3) LOC100271722 0,395 -2,249 1,26E-11 4,23E-07

21 chr14 24801501 24802001 exon exon (NM_001198592, exon 4 of 26) ADCY4 0,369 7,492 1,91E-11 6,08E-07

22 chr4 7657001 7657501 intron intron (NM_020777, intron 5 of 26) AFAP1-AS1 0,337 -2,957 2,69E-11 8,17E-07

23 chr10 16479001 16479501 intron CpG PTER 0,299 -6,968 4,89E-11 1,42E-06

24 chr9 124889001 124889501 Intergenic CpG MIR4478 0,393 4,252 5,31E-11 1,48E-06

25 chr19 42502501 42503001 3' UTR 3' UTR (NM_002088, exon 19 of 19) ATP1A3 0,465 4,240 6,35E-11 1,70E-06

26 chr16 88859501 88860001 Intergenic Intergenic PIEZO1 0,429 2,407 6,64E-11 1,71E-06

27 chr5 138729501 138730001 exon exon (NM_001161546, exon 1 of 1) C5orf65 0,385 -1,665 9,52E-11 2,36E-06

28 chr17 26874501 26875001 exon exon (NM_054035, exon 4 of 4) UNC119 0,567 2,424 1,16E-10 2,77E-06

29 chr9 19128001 19128501 promoter-TSS promoter-TSS (NM_001122) PLIN2 0,521 -2,704 1,43E-10 3,29E-06

30 chr2 85969501 85970001 Intergenic Intergenic ATOH8 0,445 -2,370 1,79E-10 3,98E-06

31 chr8 145106001 145106501 TTS TTS (NM_017570) OPLAH 0,509 4,858 2,70E-10 5,83E-06

32 chr20 24744001 24744501 Intergenic Intergenic CST7 0,525 -4,035 3,51E-10 7,34E-06

33 chr7 100167001 100167501 Intergenic CpG SAP25 0,499 7,240 5,24E-10 1,06E-05

34 chr1 156131001 156131501 exon exon (NM_001193302, exon 7 of 13) SEMA4A 0,651 -1,708 5,71E-10 1,10E-05

35 chr16 31228001 31228501 exon exon (NM_152901, exon 1 of 2) PYDC1 0,391 4,809 5,76E-10 1,10E-05

36 chr9 131013001 131013501 intron CpG-27093 MIR3154 0,429 2,498 6,26E-10 1,16E-05

37 chr3 56835501 56836001 exon exon (NM_019555, exon 1 of 10) ARHGEF3 0,293 -2,380 1,27E-09 2,27E-05

38 chr16 88700501 88701001 Intergenic CpG IL17C 0,539 3,015 1,29E-09 2,27E-05

39 chr16 31227001 31227501 TTS TTS (NM_152901) PYDC1 0,369 7,165 1,37E-09 2,35E-05

40 chr1 21009001 21009501 exon exon (NM_001122819, exon 11 of 15) DDOST 0,585 2,009 1,45E-09 2,43E-05

41 chr14 78447001 78447501 Intergenic Intergenic ADCK1 0,503 4,736 1,50E-09 2,44E-05

42 chr19 6710501 6711001 exon exon (NM_000064, exon 13 of 41) C3 0,433 2,390 1,60E-09 2,55E-05

43 chr20 3199001 3199501 non-coding non-coding (NR_052000, exon 5 of 7) ITPA 0,455 4,717 1,97E-09 3,06E-05

44 chr3 129337501 129338001 Intergenic Intergenic PLXND1 0,501 -3,651 2,13E-09 3,23E-05

# sta-arm p-value sta-arm logFC

0,469 0,465

0,229 -1,033

0,858 0,026

0,593 0,235

0,217 0,503

0,251 -0,778

0,059 1,391

0,060 1,145

0,518 -0,596

0,822 0,407

0,144 0,913

1,000 0,181

1,000 0,212

0,871 0,296

0,536 -0,331

0,003 2,440

0,858 0,073

0,075 1,485

0,649 0,878

0,295 0,789

0,426 0,802

0,128 1,567

0,346 0,239

0,475 0,715

0,914 0,084

0,884 0,087

0,334 0,986

0,961 0,257

0,073 1,330

0,075 1,384

0,426 0,479

0,469 0,326

0,612 0,376

0,884 0,334

0,511 -0,449

0,522 -0,552

0,437 0,890

0,676 0,288

0,346 0,590

0,591 -0,008

0,176 1,039

0,211 1,088

0,621 -0,012

0,217 0,764

45 chr7 26897501 26898001 intron intron (NM_003930, intron 1 of 12) SKAP2 0,485 -2,303 2,31E-09 3,41E-05

46 chr3 47050501 47051001 exon exon (NM_015175, exon 54 of 54) NRADDP 0,319 1,949 2,40E-09 3,41E-05

47 chr9 131465501 131466001 intron CpG PKN3 0,431 -4,283 2,40E-09 3,41E-05

48 chr21 47395001 47395501 Intergenic Intergenic COL6A1 0,319 2,383 2,54E-09 3,54E-05

49 chr5 140872001 140872501 TTS TTS (NM_032407) PCDHGC5 0,489 2,064 2,80E-09 3,82E-05

50 chr1 3164001 3164501 intron CpG MIR4251 0,699 1,735 2,87E-09 3,84E-05

51 chr22 23524001 23524501 exon exon (NM_004327, exon 1 of 23) BCR 0,509 -2,910 2,96E-09 3,88E-05

52 chr6 158402001 158402501 promoter-TSS promoter-TSS (NM_003898) SYNJ2 0,363 7,099 3,02E-09 3,88E-05

53 chr10 102808001 102808501 Intergenic CpG KAZALD1 0,383 1,541 3,21E-09 4,05E-05

54 chr6 163673001 163673501 intron intron (NM_001080379, intron 4 of 4) LOC285796 0,473 3,303 3,63E-09 4,50E-05

55 chr17 79428001 79428501 exon exon (NM_001080519, exon 25 of 27) MIR3186 0,387 1,552 3,80E-09 4,62E-05

56 chr9 139433001 139433501 intron intron (NM_017617, intron 2 of 33) NOTCH1 0,415 7,051 4,15E-09 4,95E-05

57 chr5 148802001 148802501 intron intron (NR_027180, intron 2 of 2) MIR143 0,327 -4,245 4,61E-09 5,40E-05

58 chr3 125709501 125710001 Intergenic Intergenic ROPN1B 0,645 2,689 5,08E-09 5,86E-05

59 chr17 29336001 29336501 Intergenic CpG DPRXP4 0,443 -1,533 5,27E-09 5,93E-05

60 chr9 111645293 111645793 intron CpG-27094 MIR3155 0,457 0,942 1,61E-08 5,66E-05

61 chr3 114195453 114195953 exon exon (NM_019555, exon 1 of 10) ARHGEF4 0,457 0,896 1,67E-08 5,83E-05

62 chr16 116745613 116746113 Intergenic CpG IL17C 0,458 0,849 1,74E-08 5,99E-05

63 chr16 119295773 119296273 TTS TTS (NM_152901) PYDC2 0,458 0,803 1,80E-08 6,16E-05

64 chr1 121845933 121846433 exon exon (NM_001122819, exon 11 of 15) DDOST 0,459 0,756 1,87E-08 6,32E-05

65 chr14 124396093 124396593 Intergenic Intergenic ADCK2 0,459 0,709 1,93E-08 6,49E-05

66 chr19 126946253 126946753 exon exon (NM_000064, exon 13 of 41) C4 0,460 0,663 1,99E-08 6,65E-05

67 chr20 129496413 129496913 non-coding non-coding (NR_052000, exon 5 of 7) ITPA 0,460 0,616 2,06E-08 6,82E-05

68 chr3 132046573 132047073 Intergenic Intergenic PLXND2 0,461 0,570 2,12E-08 6,99E-05

69 chr7 134596733 134597233 intron intron (NM_003930, intron 1 of 12) SKAP3 0,462 0,523 2,19E-08 7,15E-05

70 chr3 137146893 137147393 exon exon (NM_015175, exon 54 of 54) NRADDP 0,462 0,477 2,25E-08 7,32E-05

71 chr9 139697053 139697553 intron CpG PKN4 0,463 0,430 2,32E-08 7,48E-05

72 chr21 142247213 142247713 Intergenic Intergenic COL6A2 0,463 0,383 2,38E-08 7,65E-05

73 chr5 144797373 144797873 TTS TTS (NM_032407) PCDHGC6 0,464 0,337 2,45E-08 7,81E-05

74 chr1 147347533 147348033 intron CpG MIR4252 0,464 0,290 2,51E-08 7,98E-05

75 chr22 149897693 149898193 exon exon (NM_004327, exon 1 of 23) BCR 0,465 0,244 2,57E-08 8,14E-05

76 chr6 152447853 152448353 promoter-TSS promoter-TSS (NM_003898) SYNJ3 0,465 0,197 2,64E-08 8,31E-05

77 chr10 154998013 154998513 Intergenic CpG KAZALD2 0,466 0,150 2,70E-08 8,47E-05

78 chr6 157548173 157548673 intron intron (NM_001080379, intron 4 of 4) LOC285797 0,466 0,104 2,77E-08 8,64E-05

79 chr17 160098333 160098833 exon exon (NM_001080519, exon 25 of 27) MIR3187 0,467 0,057 2,83E-08 8,80E-05

80 chr9 162648493 162648993 intron intron (NM_017617, intron 2 of 33) NOTCH2 0,468 0,011 2,90E-08 8,97E-05

81 chr5 165198653 165199153 intron intron (NR_027180, intron 2 of 2) MIR144 0,468 -0,036 2,96E-08 9,13E-05

82 chr3 167748813 167749313 Intergenic Intergenic ROPN1B 0,469 -0,082 3,02E-08 9,30E-05

83 chr17 170298973 170299473 Intergenic CpG DPRXP5 0,469 -0,129 3,09E-08 9,46E-05

84 chr9 172849133 172849633 intron CpG-27095 MIR3156 0,470 -0,176 3,15E-08 9,63E-05

85 chr3 175399293 175399793 exon exon (NM_019555, exon 1 of 10) ARHGEF5 0,470 -0,222 3,22E-08 9,80E-05

86 chr16 177949453 177949953 Intergenic CpG IL17C 0,471 -0,269 3,28E-08 9,96E-05

87 chr16 180499613 180500113 TTS TTS (NM_152901) PYDC3 0,471 -0,315 3,35E-08 1,01E-04

88 chr1 183049773 183050273 exon exon (NM_001122819, exon 11 of 15) DDOST 0,472 -0,362 3,41E-08 1,03E-04

89 chr14 185599933 185600433 Intergenic Intergenic ADCK3 0,472 -0,408 3,48E-08 1,05E-04

0,159 1,246

0,591 -0,468

0,219 0,710

0,674 0,292

0,803 0,259

0,411 -0,358

0,397 -0,454

0,426 1,006

0,190 1,095

1,000 0,122

0,470 -0,499

0,272 -0,417

0,174 0,650

1,000 -0,032

0,032 1,933

0,444 0,397

0,444 0,398

0,444 0,399

0,444 0,400

0,444 0,402

0,444 0,403

0,445 0,404

0,445 0,405

0,445 0,406

0,445 0,407

0,445 0,409

0,445 0,410

0,445 0,411

0,445 0,412

0,446 0,413

0,446 0,414

0,446 0,416

0,446 0,417

0,446 0,418

0,446 0,419

0,446 0,420

0,446 0,421

0,447 0,423

0,447 0,424

0,447 0,425

0,447 0,426

0,447 0,427

0,447 0,428

0,447 0,430

0,447 0,431

90 chr19 188150093 188150593 exon exon (NM_000064, exon 13 of 41) C5 0,473 -0,455 3,54E-08 1,06E-04

91 chr20 190700253 190700753 non-coding non-coding (NR_052000, exon 5 of 7) ITPA 0,474 -0,502 3,60E-08 1,08E-04

92 chr3 193250413 193250913 Intergenic Intergenic PLXND3 0,474 -0,548 3,67E-08 1,10E-04

93 chr7 195800573 195801073 intron intron (NM_003930, intron 1 of 12) SKAP4 0,475 -0,595 3,73E-08 1,11E-04

94 chr3 198350733 198351233 exon exon (NM_015175, exon 54 of 54) NRADDP 0,475 -0,641 3,80E-08 1,13E-04

95 chr9 200900893 200901393 intron CpG PKN5 0,476 -0,688 3,86E-08 1,14E-04

96 chr21 203451053 203451553 Intergenic Intergenic COL6A3 0,476 -0,735 3,93E-08 1,16E-04

97 chr5 206001213 206001713 TTS TTS (NM_032407) PCDHGC7 0,477 -0,781 3,99E-08 1,18E-04

98 chr1 208551373 208551873 intron CpG MIR4253 0,477 -0,828 4,06E-08 1,19E-04

99 chr22 211101533 211102033 exon exon (NM_004327, exon 1 of 23) BCR 0,478 -0,874 4,12E-08 1,21E-04

100 chr9 213651693 213652193 intron CpG-27094 MIR3155 0,478 -0,921 4,18E-08 1,23E-04

101 chr3 216201853 216202353 exon exon (NM_019555, exon 1 of 10) ARHGEF4 0,479 -0,967 4,25E-08 1,24E-04

102 chr16 218752013 218752513 Intergenic CpG IL17C 0,480 -1,014 4,31E-08 1,26E-04

103 chr16 221302173 221302673 TTS TTS (NM_152901) PYDC2 0,480 -1,061 4,38E-08 1,28E-04

104 chr1 223852333 223852833 exon exon (NM_001122819, exon 11 of 15) DDOST 0,481 -1,107 4,44E-08 1,29E-04

105 chr14 226402493 226402993 Intergenic Intergenic ADCK2 0,481 -1,154 4,51E-08 1,31E-04

106 chr19 228952653 228953153 exon exon (NM_000064, exon 13 of 41) C4 0,482 -1,200 4,57E-08 1,33E-04

107 chr20 231502813 231503313 non-coding non-coding (NR_052000, exon 5 of 7) ITPA 0,482 -1,247 4,63E-08 1,34E-04

108 chr3 234052973 234053473 Intergenic Intergenic PLXND2 0,483 -1,294 4,70E-08 1,36E-04

109 chr7 236603133 236603633 intron intron (NM_003930, intron 1 of 12) SKAP3 0,483 -1,340 4,76E-08 1,38E-04

110 chr3 239153293 239153793 exon exon (NM_015175, exon 54 of 54) NRADDP 0,484 -1,387 4,83E-08 1,39E-04

111 chr9 241703453 241703953 intron CpG PKN4 0,484 -1,433 4,89E-08 1,41E-04

112 chr21 244253613 244254113 Intergenic Intergenic COL6A2 0,485 -1,480 4,96E-08 1,43E-04

113 chr5 246803773 246804273 TTS TTS (NM_032407) PCDHGC6 0,485 -1,526 5,02E-08 1,44E-04

114 chr1 249353933 249354433 intron CpG MIR4252 0,486 -1,573 5,09E-08 1,46E-04

115 chr22 251904093 251904593 exon exon (NM_004327, exon 1 of 23) BCR 0,487 -1,620 5,15E-08 1,48E-04

116 chr6 254454253 254454753 promoter-TSS promoter-TSS (NM_003898) SYNJ3 0,487 -1,666 5,21E-08 1,49E-04

117 chr10 257004413 257004913 Intergenic CpG KAZALD2 0,488 -1,713 5,28E-08 1,51E-04

118 chr6 259554573 259555073 intron intron (NM_001080379, intron 4 of 4) LOC285797 0,488 -1,759 5,34E-08 1,53E-04

119 chr17 262104733 262105233 exon exon (NM_001080519, exon 25 of 27) MIR3187 0,489 -1,806 5,41E-08 1,54E-04

120 chr9 264654893 264655393 intron intron (NM_017617, intron 2 of 33) NOTCH2 0,489 -1,853 5,47E-08 1,56E-04

121 chr5 267205053 267205553 intron intron (NR_027180, intron 2 of 2) MIR144 0,490 -1,899 5,54E-08 1,57E-04

122 chr3 269755213 269755713 Intergenic Intergenic ROPN1B 0,490 -1,946 5,60E-08 1,59E-04

123 chr17 272305373 272305873 Intergenic CpG DPRXP5 0,491 -1,992 5,67E-08 1,61E-04

124 chr9 274855533 274856033 intron CpG-27095 MIR3156 0,491 -2,039 5,73E-08 1,62E-04

125 chr3 277405693 277406193 exon exon (NM_019555, exon 1 of 10) ARHGEF5 0,492 -2,085 5,79E-08 1,64E-04

126 chr16 279955853 279956353 Intergenic CpG IL17C 0,493 -2,132 5,86E-08 1,66E-04

127 chr16 282506013 282506513 TTS TTS (NM_152901) PYDC3 0,493 -2,179 5,92E-08 1,67E-04

128 chr1 285056173 285056673 exon exon (NM_001122819, exon 11 of 15) DDOST 0,494 -2,225 5,99E-08 1,69E-04

129 chr14 287606333 287606833 Intergenic Intergenic ADCK3 0,494 -2,272 6,05E-08 1,71E-04

130 chr19 290156493 290156993 exon exon (NM_000064, exon 13 of 41) C5 0,495 -2,318 6,12E-08 1,72E-04

131 chr20 292706653 292707153 non-coding non-coding (NR_052000, exon 5 of 7) ITPA 0,495 -2,365 6,18E-08 1,74E-04

132 chr3 295256813 295257313 Intergenic Intergenic PLXND3 0,496 -2,412 6,24E-08 1,76E-04

133 chr7 297806973 297807473 intron intron (NM_003930, intron 1 of 12) SKAP4 0,496 -2,458 6,31E-08 1,77E-04

134 chr3 300357133 300357633 exon exon (NM_015175, exon 54 of 54) NRADDP 0,497 -2,505 6,37E-08 1,79E-04

0,447 0,432

0,448 0,433

0,448 0,434

0,448 0,435

0,448 0,437

0,448 0,438

0,448 0,439

0,448 0,440

0,448 0,441

0,449 0,443

0,449 0,444

0,449 0,445

0,449 0,446

0,449 0,447

0,449 0,448

0,449 0,450

0,449 0,451

0,450 0,452

0,450 0,453

0,450 0,454

0,450 0,455

0,450 0,457

0,450 0,458

0,450 0,459

0,450 0,460

0,451 0,461

0,451 0,462

0,451 0,464

0,451 0,465

0,451 0,466

0,451 0,467

0,451 0,468

0,451 0,469

0,452 0,471

0,452 0,472

0,452 0,473

0,452 0,474

0,452 0,475

0,452 0,476

0,452 0,478

0,452 0,479

0,452 0,480

0,453 0,481

0,453 0,482

0,453 0,483

135 chr9 302907293 302907793 intron CpG PKN5 0,497 -2,551 6,44E-08 1,81E-04

136 chr21 305457453 305457953 Intergenic Intergenic COL6A3 0,498 -2,598 6,50E-08 1,82E-04

137 chr5 308007613 308008113 TTS TTS (NM_032407) PCDHGC7 0,499 -2,644 6,57E-08 1,84E-04

138 chr1 310557773 310558273 intron CpG MIR4253 0,499 -2,691 6,63E-08 1,86E-04

139 chr22 313107933 313108433 exon exon (NM_004327, exon 1 of 23) BCR 0,500 -2,738 6,70E-08 1,87E-04

140 chr6 315658093 315658593 promoter-TSS promoter-TSS (NM_003898) SYNJ4 0,500 -2,784 6,76E-08 1,89E-04

141 chr10 318208253 318208753 Intergenic CpG KAZALD3 0,501 -2,831 6,82E-08 1,91E-04

142 chr6 320758413 320758913 intron intron (NM_001080379, intron 4 of 4) LOC285798 0,501 -2,877 6,89E-08 1,92E-04

143 chr17 323308573 323309073 exon exon (NM_001080519, exon 25 of 27) MIR3188 0,502 -2,924 6,95E-08 1,94E-04

144 chr9 325858733 325859233 intron intron (NM_017617, intron 2 of 33) NOTCH3 0,502 -2,970 7,02E-08 1,95E-04

145 chr5 328408893 328409393 intron intron (NR_027180, intron 2 of 2) MIR145 0,503 -3,017 7,08E-08 1,97E-04

146 chr3 330959053 330959553 Intergenic Intergenic ROPN1B 0,503 -3,064 7,15E-08 1,99E-04

147 chr17 333509213 333509713 Intergenic CpG DPRXP6 0,504 -3,110 7,21E-08 2,00E-04

148 chr9 336059373 336059873 intron CpG-27096 MIR3157 0,505 -3,157 7,28E-08 2,02E-04

149 chr3 338609533 338610033 exon exon (NM_019555, exon 1 of 10) ARHGEF6 0,505 -3,203 7,34E-08 2,04E-04

150 chr11 33890501 33891001 intron CpG LMO2 0,451 -6,228 3,52E-07 1,57E-03

151 chr11 61596501 61597001 intron intron (NM_004265, intron 1 of 11) FADS2 0,361 -6,226 3,57E-07 1,58E-03

152 chr1 201619001 201619501 intron CpG NAV1 0,625 1,255 3,84E-07 1,69E-03

153 chr20 9489001 9489501 Intergenic Intergenic LAMP5 0,339 -3,890 3,94E-07 1,72E-03

154 chr5 177783501 177784001 intron intron (NM_173465, intron 2 of 28) AGXT2L2 0,373 -1,908 4,02E-07 1,74E-03

155 chr6 150285501 150286001 intron CpG ULBP1 0,473 -3,268 4,04E-07 1,74E-03

156 chr4 147558001 147558501 Intergenic CpG POU4F2 0,355 6,602 4,05E-07 1,74E-03

157 chr15 20990501 20991001 Intergenic CpG NBEAP1 0,377 4,262 4,08E-07 1,74E-03

158 chr8 42052501 42053001 intron intron (NM_033011, intron 1 of 12) PLAT 0,351 -3,882 4,21E-07 1,78E-03

159 chr12 49487501 49488001 intron intron (NM_021044, intron 1 of 2) DHH 0,263 6,596 4,23E-07 1,78E-03

160 chr16 216001 216501 intron CpG-8387 HBM 0,425 -6,192 4,32E-07 1,80E-03

161 chr19 40871501 40872001 5' UTR 5' UTR (NM_012268, exon 2 of 13) C19orf47 0,483 1,547 4,40E-07 1,82E-03

162 chr1 878001 878501 exon exon (NM_152486, exon 11 of 14) NOC2L 0,731 3,216 4,41E-07 1,82E-03

163 chr19 39755501 39756001 Intergenic CpG IL28A 0,379 1,913 4,44E-07 1,82E-03

164 chr1 86043501 86044001 5' UTR 5' UTR (NM_001134445, exon 1 of 7) DDAH1 0,551 2,398 4,57E-07 1,86E-03

165 chr17 76136501 76137001 intron CpG C17orf99 0,351 6,595 4,61E-07 1,87E-03

166 chr12 133177501 133178001 Intergenic Intergenic LOC100507055 0,427 2,114 4,66E-07 1,87E-03

167 chr11 281001 281501 exon exon (NM_138329, exon 4 of 8) NLRP6 0,273 3,206 4,66E-07 1,87E-03

168 chrX 70586001 70586501 exon exon (NM_004606, exon 1 of 38) TAF1 0,609 -1,316 4,88E-07 1,94E-03

169 chr15 96952501 96953001 Intergenic CpG NR2F2 0,367 3,202 5,08E-07 2,01E-03

170 chr16 67199501 67200001 intron CpG-9393 HSF4 0,218 2,235 5,24E-07 2,06E-03

171 chr12 31270501 31271001 Intergenic Intergenic LOC100506660 0,613 6,564 5,72E-07 2,24E-03

172 chr17 40932501 40933001 exon exon (NM_032387, exon 1 of 19) WNK4 0,461 3,571 5,81E-07 2,26E-03

173 chr19 42420001 42420501 Intergenic Intergenic ARHGEF1 0,387 1,852 5,90E-07 2,28E-03

174 chr19 44038001 44038501 intron CpG-13861 ZNF575 0,373 6,568 5,99E-07 2,30E-03

175 chr14 105714501 105715001 promoter-TSS promoter-TSS (NM_145685) BTBD6 0,321 6,565 6,11E-07 2,34E-03

176 chr17 43508001 43508501 intron CpG ARHGAP27 0,275 2,215 6,17E-07 2,35E-03

177 chr2 242742501 242743001 intron CpG NEU4 0,230 1,499 6,47E-07 2,45E-03

178 chr11 20153501 20154001 Intergenic L1M3f|LINE|L1 DBX1 0,391 -3,210 6,61E-07 2,48E-03

179 chr3 47051001 47051501 TTS TTS (NM_015175) NRADDP 0,267 1,591 6,72E-07 2,51E-03

0,453 0,485

0,453 0,486

0,453 0,487

0,453 0,488

0,453 0,489

0,454 0,490

0,454 0,492

0,454 0,493

0,454 0,494

0,454 0,495

0,454 0,496

0,454 0,497

0,454 0,499

0,455 0,500

0,455 0,501

0,612 0,392

0,144 0,530

0,923 -0,346

0,346 0,239

0,497 0,608

0,144 0,862

1,000 -0,021

0,658 -0,018

0,469 0,419

0,196 0,930

0,023 0,834

0,847 -0,007

0,048 0,960

0,311 0,916

0,060 1,157

0,666 -0,129

0,585 0,364

0,822 0,313

0,201 0,814

0,005 1,585

0,297 0,941

0,330 -0,367

0,272 0,677

0,805 -0,170

0,426 0,701

0,346 0,511

0,318 -0,938

0,771 0,180

0,196 0,753

0,190 -0,999

180 chr5 139927001 139927501 promoter-TSS promoter-TSS (NM_003732) EIF4EBP3 0,321 6,551 6,75E-07 2,51E-03

181 chr8 145638501 145639001 exon exon (NM_017767, exon 9 of 11) SLC39A4 0,347 3,173 6,80E-07 2,51E-03

182 chr17 47297001 47297501 exon exon (NM_001135186, exon 5 of 8) GNGT2 0,407 1,241 6,83E-07 2,51E-03

0,409 -0,238

0,897 0,335

0,736 -0,297

183 chr9 129885001 129885501 promoter-TSS promoter-TSS (NM_012098) ANGPTL2 0,559 4,215 6,86E-07 2,51E-03 NA 0,114

184 chr22 20232501 20233001 intron intron (NM_023004, intron 1 of 1) MIR1286 0,543 6,532 7,12E-07 2,59E-03

185 chr1 235254001 235254501 Intergenic Intergenic SNORA14B 0,535 -1,701 7,33E-07 2,64E-03

186 chr5 140871501 140872001 TTS TTS (NM_032407) PCDHGC5 0,401 4,215 7,34E-07 2,64E-03

187 chr11 640001 640501 exon exon (NM_000797, exon 3 of 4) DRD4 0,405 3,160 7,66E-07 2,74E-03

188 chr22 23523501 23524001 exon exon (NM_004327, exon 1 of 23) BCR 0,515 -3,475 7,76E-07 2,76E-03

189 chr16 2907501 2908001 intron CpG PRSS22 0,435 1,777 8,00E-07 2,83E-03

190 chr5 1594501 1595001 promoter-TSS promoter-TSS (NR_003263) SDHAP3 0,631 -2,021 8,23E-07 2,90E-03

191 chr16 629001 629501 intron intron (NM_148920, intron 7 of 9) PIGQ 0,307 4,191 8,34E-07 2,92E-03

192 chr2 241853501 241854001 Intergenic MIRc|SINE|MIR C2orf54 0,383 2,072 8,66E-07 3,01E-03

193 chr5 76115501 76116001 intron CpG F2RL1 0,329 -1,935 8,70E-07 3,01E-03

194 chr11 70455001 70455501 intron intron (NM_133266, intron 2 of 10) SHANK2-AS1 0,431 1,221 9,15E-07 3,15E-03

195 chr2 233981501 233982001 intron intron (NM_001017915, intron 2 of 25) INPP5D 0,395 1,297 9,19E-07 3,15E-03

196 chr9 131012501 131013001 intron CpG MIR3154 0,491 2,346 9,43E-07 3,21E-03

197 chr2 235860001 235860501 promoter-TSS promoter-TSS (NM_014521) SH3BP4 0,391 -6,116 9,46E-07 3,21E-03

198 chr5 41869501 41870001 intron CpG OXCT1 0,303 -1,535 9,71E-07 3,28E-03

199 chr3 49170501 49171001 promoter-TSS promoter-TSS (NM_002292) LAMB2 0,441 -3,191 9,79E-07 3,29E-03

200 chr20 23211501 23212001 Intergenic Intergenic LOC200261 0,413 -2,533 9,86E-07 3,30E-03

201 chr16 57079501 57080001 intron intron (NM_032206, intron 20 of 47) NLRC5 0,485 1,576 1,05E-06 3,49E-03

202 chr3 128210501 128211001 intron CpG-18893 GATA2 0,357 -3,435 1,07E-06 3,53E-03

203 chr8 72754501 72755001 promoter-TSS promoter-TSS (NR_033652) LOC100132891 0,509 3,509 1,08E-06 3,54E-03

204 chr16 67199001 67199501 intron CpG HSF4 0,255 2,343 1,10E-06 3,54E-03

205 chr1 2130501 2131001 non-coding non-coding (NR_046424, exon 4 of 8) C1orf86 0,657 -2,849 1,10E-06 3,54E-03

0,256 -0,359

0,108 1,430

0,284 0,602

0,611 0,083

0,300 -0,417

0,497 0,351

0,024 1,410

0,866 0,349

0,110 1,139

0,747 0,838

0,563 0,454

0,809 0,021

0,277 -0,689

0,024 1,006

0,566 0,653

0,932 0,201

0,794 0,361

0,923 0,459

0,196 0,753

0,426 0,851

0,032 1,302

0,009 1,256

206 chr7 149487001 149487501 intron intron (NM_198455, intron 31 of 109) SSPO 0,289 6,479 1,10E-06 3,54E-03 NA 0,114

207 chr1 1182001 1182501 promoter-TSS promoter-TSS (NM_001014980) FAM132A 0,772 2,697 1,10E-06 3,54E-03

208 chr3 158449501 158450001 intron intron (NM_002888, intron 1 of 3) RARRES1 0,465 4,164 1,11E-06 3,55E-03

0,270 0,553

0,196 0,677

209 chr3 182817001 182817501 5' UTR 5' UTR (NM_020166, exon 1 of 19) MCCC1 0,369 -6,086 1,11E-06 3,55E-03 NA 0,114

210 chr6 113886001 113886501 Intergenic Intergenic MARCKS 0,461 -1,663 1,13E-06 3,61E-03 0,698 0,437
